# Neurocalcin Delta Knockout Impairs Adult Neurogenesis Whereas Half Reduction Is Not Pathological

**DOI:** 10.3389/fnmol.2019.00019

**Published:** 2019-02-12

**Authors:** Aaradhita Upadhyay, Seyyedmohsen Hosseinibarkooie, Svenja Schneider, Anna Kaczmarek, Laura Torres-Benito, Natalia Mendoza-Ferreira, Melina Overhoff, Roman Rombo, Vanessa Grysko, Min Jeong Kye, Natalia L. Kononenko, Brunhilde Wirth

**Affiliations:** ^1^Institute of Human Genetics, University of Cologne, Cologne, Germany; ^2^Center for Molecular Medicine Cologne, University of Cologne, Cologne, Germany; ^3^Institute for Genetics, University of Cologne, Cologne, Germany; ^4^Excellence Cluster on Cellular Stress Responses in Aging Associated Diseases (CECAD), University of Cologne, Cologne, Germany; ^5^Center for Rare Diseases Cologne, University Hospital of Cologne, Cologne, Germany

**Keywords:** neurocalcin delta, neuronal calcium sensor, adult neurogenesis, MAP3K10, pJNK activation, spinal muscular atrophy, survival motor neuron

## Abstract

Neurocalcin delta (NCALD) is a brain-enriched neuronal calcium sensor and its reduction acts protective against spinal muscular atrophy (SMA). However, the physiological function of NCALD and implications of NCALD reduction are still elusive. Here, we analyzed the ubiquitous *Ncald* knockout in homozygous (*Ncald*^KO/KO^) and heterozygous (*Ncald*^KO/WT^) mice to unravel the physiological role of NCALD in the brain and to study whether 50% NCALD reduction is a safe option for SMA therapy. We found that *Ncald*^KO/KO^ but not *Ncald*^KO/WT^ mice exhibit significant changes in the hippocampal morphology, likely due to impaired generation and migration of newborn neurons in the dentate gyrus (DG). To understand the mechanism behind, we studied the NCALD interactome and identified mitogen-activated protein kinase kinase kinase 10 (MAP3K10) as a novel NCALD interacting partner. MAP3K10 is an upstream activating kinase of c-Jun N-terminal kinase (JNK), which regulates adult neurogenesis. Strikingly, the JNK activation was significantly upregulated in the *Ncald*^KO/KO^ brains. Contrary, neither adult neurogenesis nor JNK activation were altered by heterozygous *Ncald* deletion. Taken together, our study identifies a novel link between NCALD and adult neurogenesis in the hippocampus, possibly *via* a MAP3K10-JNK pathway and emphasizes the safety of using NCALD reduction as a therapeutic option for SMA.

## Introduction

Neurocalcin delta (NCALD) is a brain-enriched highly conserved neuronal calcium sensor protein (Wang et al., 2001; Di Sole et al., 2012). Recently, we have shown that reduced NCALD levels protect against spinal muscular atrophy (SMA) in individuals carrying homozygous deletion of *SMN1* and only four *SMN2* copies (Riessland et al., 2017). In that study, five *SMN1*-deleted individuals from a large SMA family were asymptomatic while two were symptomatic. Asymptomatic individuals showed a reduction of NCALD of approximately 50% in fibroblast and almost 80% in lymphoblastoid cell lines in comparison to affected individuals (Riessland et al., 2017). SMA is one of the most common autosomal recessive disorders in humans and the most common genetic cause of early childhood lethality (Mercuri et al., 2018b). Usually, four *SMN2* copies in the presence of homozygous deletion of *SMN1* result in mild type III SMA (Wirth et al., 2006). The most severely affected cells in SMA patients are spinal motor neurons (MNs), whose loss causes muscle weakness and atrophy (Finkel et al., 2018; Mercuri et al., 2018b). Moreover, we found that reduced NCALD levels ameliorate MN defects also in genetically modified SMA animal models (worm, zebrafish and mice), indicating that NCALD reduction acts SMA protective across species (Riessland et al., 2017). Strategies to treat SMA include the splicing modulation of the *SMN2* copy gene or gene replacement therapy (Finkel et al., 2017; Mendell et al., 2017). Recently, Nusinersen, an antisense oligonucleotide (ASO) that restores the *SMN2* splicing has been approved by the US Food and Drug Administration (FDA) and European Medicines Agency (EMA) as a first drug treatment for SMA (Finkel et al., 2017; Hoy, 2017). However, since 60% of SMA patients usually carry only two *SMN2* copies and develop the severe form of SMA, augmenting the SMN level solely *via* splice correction molecules seems to be insufficient to cure SMA (Finkel et al., 2017; Mercuri et al., 2018a). Therefore, targeting additional SMN-independent pathways that support the MN function—such as NCALD reduction—are urgently needed (Wirth et al., 2015).

While acting protective in SMA, NCALD reduction has also been associated with various neurological disorders. NCALD levels are downregulated in the brains of patients with Alzheimer’s disease (Shimohama et al., 1996; Miller et al., 2013) and in a genetic mouse model of schizophrenia (Vercauteren et al., 2007). Additionally, single nucleotide polymorphisms (SNPs) in *NCALD* have been associated with autism and bipolar disorder (Ben-David et al., 2011; Xu et al., 2014).

NCALD is a member of visinin-like proteins (VSNLs) subfamily of neuronal calcium sensors, which includes the additional four members VILIP1, VILIP2, VILIP3 and hippocalcin (Braunewell and Klein-Szanto, 2009). Depending on their location within the cell and interactions with other proteins, VSNLs transduce the Ca^2+^ signals into specific cellular changes (Burgoyne, 2007; Braunewell and Klein-Szanto, 2009). NCALD, like the other VSNLs, possesses three functional EF hand motifs, which upon Ca^2+^ binding cause the extrusion of myristoyl chain and enables NCALD for insertion into the biological membranes. Cytoplasmic myristoylated NCALD can interact with outer mitochondrial membrane and endoplasmic reticulum (ER; Iino et al., 1995; Ladant, 1995). Furthermore, NCALD has been reported to interact with microsomal cytochrome b5 (Cyb5) on the ER membrane and modulate NADH-dependent microsomal electron transport pathway (Oikawa et al., 2016).

Moreover, NCALD has been found to interact with actin and clathrin, both proteins essential for endocytosis (Ivings et al., 2002; Riessland et al., 2017). Accordingly, NCALD is implicated in the regulation of multiple endocytosis-dependent neuronal functions, like neurotransmitter release, axonal growth and branching (Vercauteren et al., 2007; Yamatani et al., 2010; Riessland et al., 2017). In MN-like cells, calcium influx is reduced which facilitates the binding of NCALD to clathrin. Consequently, NCALD reduction releases clathrin and thus, allows its function in vesicle coating restoring impaired endocytosis in SMA (Riessland et al., 2017).

In conclusion, NCALD reduction acts protective in SMA and at the same time is associated with various neurological diseases. Hence, this study aims to provide an insight into the pathophysiology of homozygous and heterozygous *Ncald* deletion in the brain. To understand the function of NCALD in the brain and to unravel the physiological consequences of its reduction for SMA therapy, we characterized the NCALD depletion in the mouse central nervous system (CNS), using conventional *Ncald* knockout mice from Jackson laboratory (Stock No 018575).

## Materials and Methods

### Mouse Experiments

All animal procedures were conducted in accordance with European, national and institutional guidelines and protocols, and were approved by the responsible government authority: Landesamt für Natur, Umwelt und Verbraucherschutz NRW (Animal Licence: LANUV NRW under the reference number 84-02.04.2014.A 126). Homozygous *Ncald*^KO/KO^ and heterozygous *Ncald*^KO/WT^ [B6N(Cg)-*Ncald*^tm1.1(KOMP)Vlcg^/J, Stock Number: 018575] animals were acquired from Jackson Laboratory. Animals used for all experiments were provided food and water *ad libitum* and were caged in small groups on a 12 h light/ dark cycle. These animals were genotyped using following primers: mmu *Ncald*WTfw: 5′-TTTCCCTTACGGGGATGCT-3′; mmu *Ncald*WTrev: 5′-AGCATTTCTGCCTTGCTGAT-3′; mmu *Ncald*KOfw: 5′-CGGTCGCTACCATTAC-3′; mmu *Ncald*KOrev: 5′-GCATGTGTGACAACAG-3′.

### Western Blot Analysis

Tissues were lysed in ice cold RIPA buffer (Sigma) together with protease (Complete Mini, Roche) and phosphatase inhibitors (Thermo scientific, Waltham, MA, USA). For further analysis, the following primary antibodies were used; anti-NCALD (1:1,000, 12925-1-AP, Proteintech), anti-beta-actin (1:2,500, A5316, Sigma), anti-GAPDH (1:5,000 G-9295, Sigma), anti-myelin basic protein (anti-MBP; 1:1,000 SMI94, Covance), anti- MAP3K10 (1:500, NBP1-87737, Novus Biologicals), anti-pJNK (1:500, sc-6254, Santa Cruz), anti-JNK (1:1,000, #9252, Cell signaling). Chemiluminescence signal was detected with HRP conjugated-secondary antibodies and Chemiluminescence reagent (Thermo Scientific, Waltham, MA, USA) according to manufacturer’s protocol.

### Nissl Staining

A freezing microtome was used for cutting 40 μm thick consecutive horizontal brain sections. 0.2% gelatin solution in 250 mM Tris-HCl was used for mounting the sections and left overnight at 40°C heating plate for drying. For further staining the sections were first rehydrated for 1 min in water and then stained in 0.1% cresyl violet solution for approximately 8 min. Following this, the sections were washed three times in water (2 min each) and an ascending ethanol series (50%, 70%, 80%, 90%) was used for dehydration. Sections were finally destained with 96% ethanol and 0.5% acetic acid solution and washed twice in 100% ethanol (2 min each). Subsequently, the sections were incubated in xylene for at least 2 min or until they were mounted using Entellan.

### Immunohistochemical Analysis of Brain Sections

Mice were sacrificed at P14, P30 or 4 months by ketamine/ xylazine overdose followed by transcardial perfusion with saline solution (0.85% NaCl, 0.025% KCl, 0.02% NaHCO_3_, pH 6.9, 0.01% heparin, body temperature) and ice cold 4% paraformaldehyde (PFA) freshly depolymerized in 1×phosphate-buffered saline (PBS), pH 7.4. The fixed brains were carefully isolated from the skull and were further stored overnight in the same ice-cold 4% PFA solution as used for transcardial perfusion. For further storage and cryoprotection, the brains were transferred to a mixture of 20% glycerol and 2% dimethylsulfoxide in 0.1 M phosphate buffer. Consecutive horizontal sections (40 μm) were collected in six series using a freezing microtome. Corresponding brain sections from wildtype (WT) and *Ncald*^KO/KO^ littermates (gender matched) were stained simultaneously for further immunohistochemical analysis as previously described (Kononenko et al., 2017). The following antibodies were used: anti-NCALD (1:100, 12925-1-AP, Proteintech), anti-NeuN (1:500, EPR 12763, Abcam), anti-TBR1 (1:500, ab31940), anti-CUX1 (1:200 sc-13024, Santa Cruz), anti-glial fibrillary acidic protein (anti-GFAP; 1:500, G3893, Sigma), anti-Ki-67 (1:500, ab15580 Abcam), anti-DCX (1:500, AB2253, Merck), anti-adenomatous polyposis coli (anti-APC; 1:500, OP80, Merck), anti-MBP (1:1,000 SMI94, Covance).

### Hippocampal Neuronal Culture and Sholl Analysis

Hippocampi were isolated from P1–P5 postnatal mice and the neurons were cultured as described previously (Kononenko et al., 2013). Calcium phosphate transfection procedure was used to transfect the cultured hippocampal neurons at DIV 6–8 with eGFP plasmid as previously described (Threadgill et al., 1997). Neurons were fixed using 4% PFA on DIV 14–15 and were additionally immunostained with anti-GFP (1:5,000, ab13970, Abcam) antibody. Neurons were then imaged with AxioImager M2 fluorescence microscope (Zeiss) appended with ApoTome.2 which allowed virtual confocality. Single cells captured with soma in the center of the image were then subjected to Sholl analysis using ImageJ Sholl Analysis Macro (Gensel et al., 2010). Dendritic branching was determined by adding the total number of intersections within 220 μm from the cell body.

### Primary Motor Neuron Culture

E13.5 mouse embryos were used for dissecting spinal cords (Hosseinibarkooie et al., 2016). Trypsin (Worthington) and DNAse (Sigma) mixture was used for isolating neurons. These singularized neurons were sieved and plated on poly-D-lysine/laminin (Sigma) coated coverslips. Neurobasal medium along with B27 supplement, 2 mM L-glutamine, 1× pen-strep (Invitrogen, Carlsbad, CA, USA) containing 50 ng/μl BDNF, 50 ng/μl GCNF and 50 ng/μl CNTF (Peprotech) was used as culture medium at 37°C in a humidified incubator with 5% CO_2_ add space after 5%). Along with axonal length (longest neurite), we have also analyzed primary branching and secondary branching (branches from the longest neurite were considered primary whereas branches from the primary branches were considered secondary) of these MNs.

### Immunocytochemistry for Cultured Neurons

On DIV 14 the neurons on coverslips were fixed using 4% PFA in PBS at RT for 15 min and washed three times with 1×PBS at room temperature. After blocking with PBS containing 5% normal goat serum (NGS) and 0.3% Saponin for 1 h, neurons were incubated for 1 h with following primary antibodies anti-NCALD (1:600, 12925-1-AP), anti-VGLUT1 (1:300, 131004, Synaptic Systems), anti-VGAT (1:300, 135011, Synaptic Systems), anti-TAU (1:800, sc-390476, Santa Cruz) and anti-Choline Acetyltransferase (anti-CHAT; 1:500, AB144P, Millipore, Burlington MA, USA) diluted in blocking solution. Coverslips with neurons were then rinsed three times with PBS and incubated for 30 min with corresponding secondary antibodies (diluted 1:1,000 in PBS containing 0.3% Saponin and 5% NGS). Subsequently, coverslips with neurons were washed three times in 1×PBS and mounted using Immumount. A random stretch of neurites with certain observable puncta with co-localization (yellow) signal was chosen. A line was drawn on this stretch and ImageJ plot profile function was used for each channel individually to calculate the intensity plot along the line. Intensity values were plotted against the XY value on the line for each channel in GraphPad Prism 6 software. Subsequently, the plots for NCALD and each synaptic marker were superimposed. The asterisks represent overlapping peaks of each channel, thus showing the co-localization.

### Confocal Microscopy

Confocal imaging was performed using a commercial Leica SP8 TCS microscope (Leica Microsystems) equipped with four laser lines 405 nm, 488 nm, 552 nm and 638 nm. Samples within each independent experiment were acquired with equal settings. Images were acquired with an HC PL APO 20×/0.75 CS2 or HC PL APO 63×/1.40 CS2 (oil) objectives (Leica Microsystems), a scanning format of 1,024 × 1,024, eight-bit sampling, and 1 zoom, yielding a pixel dimension of 567.62 × 567.62 or 90.09 nm × 90.09 nm in the *x* and *y* dimensions, respectively.

### Co-immunoprecipitation

The brain and spinal cord samples were collected at P30 and P14, respectively (From both WT and *Ncald*^KO/KO^ mice). The tissue samples were homogenized and lysed in NP40-based lysis buffer (50 mM Tris-HCl, 1% NP40, 100 mM NaCl, 2 mM MgCl2 including protease inhibitor). Ten microliter of Control rabbit IgG (SantaCruz) and NCALD polyclonal antibody were used for immunoprecipitation using protein A paramagnetic MicroBeads (Miltenyi) following the manufacturer’s instruction. Finally, the IP columns were washed at least six times with lysis buffer. The bound fraction of proteins was directly used for further mass spectrometry analysis.

### Mass Spectrometry and Data Analysis

All samples were analyzed on a Q-Exactive Plus (Thermo Scientific, Waltham, MA, USA) mass spectrometer coupled to an EASY nLC 1,000 UPLC (Thermo Scientific, Waltham, MA, USA). Peptides were loaded with solvent A (0.1% formic acid in water) onto an in-house packed analytical column (50 cm × 75 μm I.D., filled with 2.7 μm Poroshell EC120 C18, Agilent). Peptides were chromatographically separated at a constant flow rate of 250 nL/min using the following gradient: 5–30% solvent B (0.1% formic acid in 80% acetonitrile) within 66 min, 30–50% solvent B within 13 min, followed by washing and column equilibration. The mass spectrometer was operated in data-dependent acquisition mode. The MS1 survey scan was acquired from 300 to 1,750 m/z at a resolution of 70,000. The top 10 most abundant peptides were isolated within a 1.8 Th window and subjected to HCD fragmentation at a normalized collision energy of 27%. The AGC target was set to 5e5 charges, allowing a maximum injection time of 120 ms. Product ions were detected in the Orbitrap at a resolution of 35,000. Precursors were dynamically excluded for 20 s.

All mass spectrometric raw data were processed with Maxquant (version 1.5.3.8) using default parameters. Briefly, MS2 spectra were searched against the Uniprot MOUSE.fasta (downloaded at: 18.47.2017) database, including a list of common contaminants. False discovery rates on protein and PSM level were estimated by the target-decoy approach to 1% (Protein FDR) and 1% (PSM FDR) respectively. The minimal peptide length was set to seven amino acids and carbamidomethylation at cysteine residues was considered as a fixed modification. Oxidation (M) and Acetyl (Protein N-term) were included as variable modifications. The match-between runs option was enabled. LFQ quantification was enabled using default settings.

### Statistical Analysis

Statistical significance was calculated from independent experiments (*n*) for analysis of all experiments. A two-tailed unpaired student’s *t*-test was used to evaluate the statistical significance between two groups for all normally distributed raw data. The statistical significance between more than two groups for all normally distributed raw data was evaluated using one-way analyses of variance (ANOVA; Tukey *post hoc* test was used to determine the statistical significance between the groups). Significant differences were accepted at *p* < 0.05. For box plots the median divides the box, while the upper boundary of the box corresponds to the third quartile and the lower boundary corresponds to the first quartile. The minimum and the maximum values extend as bars from the bottom and top of the box.

## Results

### Homozygous Loss of *Ncald* Alters Gross Morphology of the Brain

**Figure 1:**
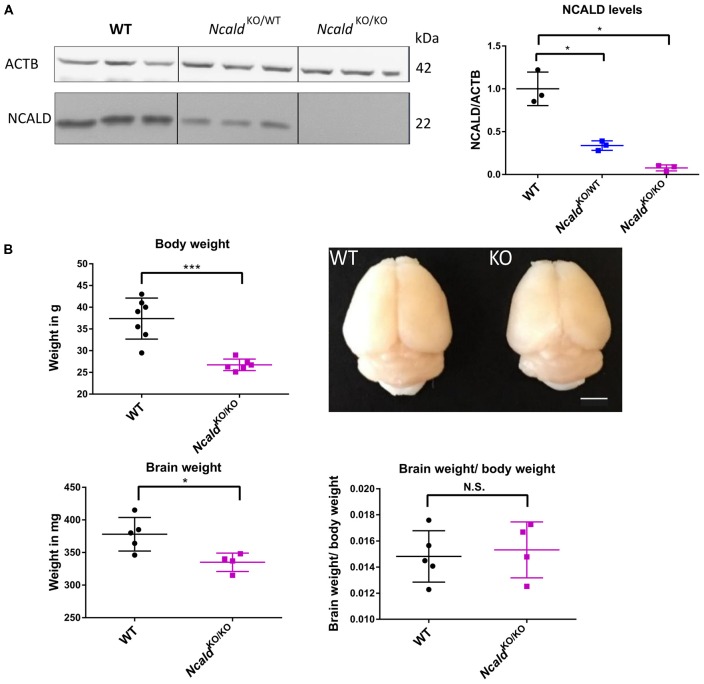
Neurocalcin delta (*Ncald*)^KO/KO^ animals weight less and have smaller brains than wildtype (WT) littermates.** (A)** Western blot analysis of brain lysates from WT, *Ncald*^KO/WT^ and *Ncald*^KO/KO^ mice showing a significant reduction of NCALD in *Ncald*^KO/WT^ animals and absence of NCALD in *Ncald*^KO/KO^ animals; *P > 0.05. **(B)** Body weight of 5-month-old *Ncald*^KO/KO^ males is significantly reduced (*N* = 6) compared to their WT littermates (*N* = 7); *P* < 0.05. Representative images of 4-month-old *Ncald*^KO/KO^ (*N* = 4) and WT (*N* = 5) littermate brains and dot plot quantifications, indicating significantly smaller brains, but no significant changes in brain-to-body mass ratio in *Ncald*^KO/KO^ animals compared to WT littermates; scale bar 100 pixels; *P < 0.05; ***P < 0.005; N.S. = not significant. Uncropped Western blots are included in Supplementary Data Sheet 8.

In order to gain an in-depth understanding of NCALD function in the brain, we acquired the heterozygous *Ncald* knockout (*Ncald*^KO/WT^) animals from the Jackson Laboratories [Bl6N(Cg)-*Ncald*^tm1.1(KOMP)Vlcg^/J, Stock Number: 018575]. After crossing the heterozygous parents, we obtained 25% *Ncald*^KO/KO^, 50% *Ncald*^KO/WT^ and 25% *Ncald*^WT/WT^ mice, according to the Mendelian inheritance. *Ncald*^KO/KO^ mice were fertile (with lower fertility rate) and showed normal survival (data not shown). We observed more than a half of NCALD reduction in *Ncald*^KO/WT^ and a complete absence of NCALD in *Ncald*^KO/KO^ animals in the brain (Figure 1A). Furthermore, *Ncald*^KO/KO^ animals revealed significantly reduced body weight compared to their WT littermates (Figure 1B). Since NCALD is highly abundant in neurons, we next analyzed the gross morphology of NCALD deficient brains (Figure 1B). We found that *Ncald*^KO/KO^ animals have significantly smaller brains compared to WT littermates, however when normalized to reduced body weight the reduction in brain weight was not significant (Figure 1B). Interestingly, neither the body weight nor the brain weight or morphology were altered by the heterozygous *Ncald* knockout (Supplementary Figures S1A–C).

**Figure 2:**
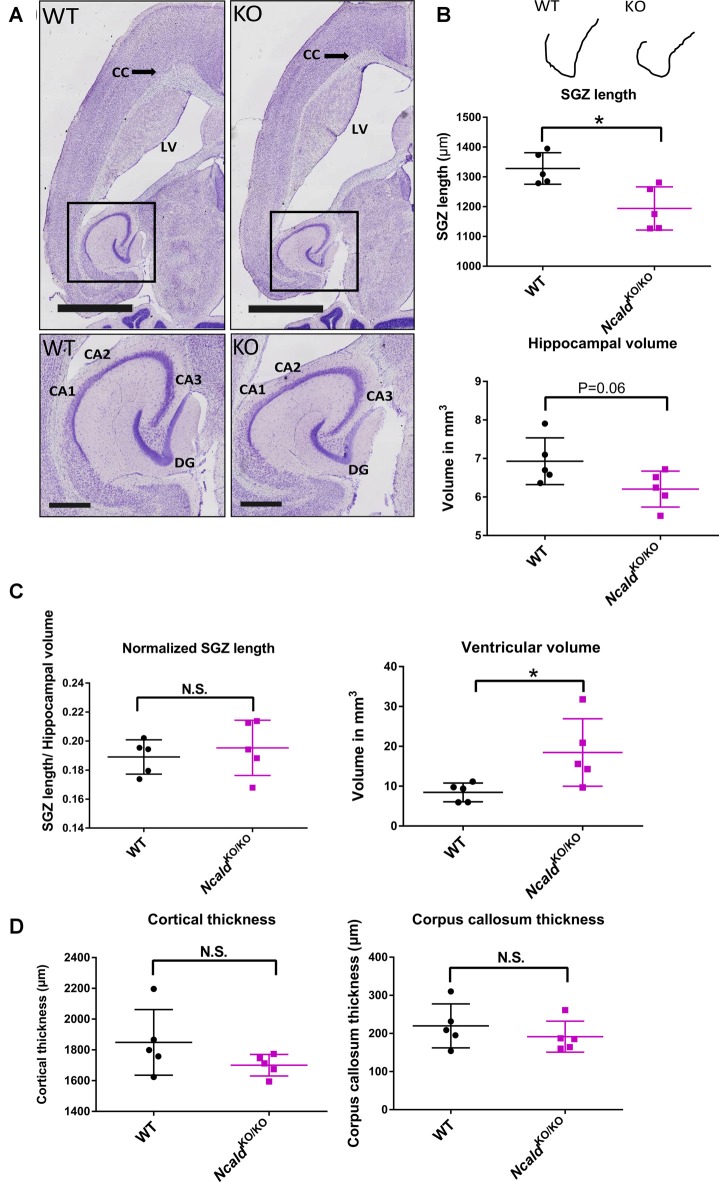
*Ncald*^KO/KO^ mice exhibit abnormal brain gross morphology when compared to WT littermates. **(A)** Representative examples of Nissl-stained 4-month-old *Ncald*^KO/KO^ and WT brains; scale bars 2 mm and 500 μm (magnified inset). **(B)** Schematic illustration of a tracing line used to manually measure the subgranular zone (SGZ) length on Nissl-stained consecutive brain section. Dot plots representing a reduction in the SG length (SGL) of the dentate gyrus (DG) and a strong tendency towards a smaller hippocampal volume in *Ncald*^KO/KO^ mice compared to WT littermates; *N* = 5; *P < 0.05. **(C)** Dot plots representing no significant difference in the SGL, when normalized to the hippocampal volume and significantly increased volume of lateral ventricles in *Ncald*^KO/KO^ mice compared to WT littermates; *N* = 5; *P < 0.05; N.S. = not significant. **(D)** Dot plots representing unaltered cortical thickness and corpus callosum thickness in the *Ncald*^KO/KO^ animals in comparison to WT littermates; *N* = 5; N.S. = not significant.

Next, we used the Nissl staining on consecutive 40 μm thin sections to characterize the brain morphology of *Ncald*^KO/KO^ mice at 2 weeks (P14), 1 month (P30) and 4-months of age (adult). We found that although no significant changes in brain weight and gross morphology could be detected at P14 (Supplementary Figures S2A,B) and P30 (Supplementary Figures S2C,D), adult *Ncald*^KO/KO^ brains exhibited significantly enlarged lateral ventricles and disturbed hippocampal morphology, which was accompanied by a significantly reduced subgranular zone length (SGZ; Figures 2A–D). Interestingly, this SGZ reduction was proportional to the hippocampal volume, indicating that *Ncald*^KO/KO^ brains possess smaller hippocampi in general. These morphological changes in *Ncald*^KO/KO^ animals could either indicate a brain maturation defect occurring during the adolescence or be a sign of a progressive neurodegeneration, which would get more severe later in the adulthood. To answer this question we analyzed the brain morphology of 1.5 years *Ncald*^KO/KO^ animals and older. However, we did not detect any severe exacerbation of the phenotype in old *Ncald*^KO/KO^ animals compared to the adult mice (data not shown). *Ncald*^KO/KO^ brains also revealed no signs of astrogliosis, marked by immunostaining for GFAP (Pekny and Pekna, 2014; Supplementary Figure S3A). Furthermore, neither overall neuronal cell density nor neuronal complexity *per se* were altered by the homozygous *Ncald* knockout in mice (Supplementary Figures S3B,C). Taken together, our data point to brain maturation defects in the adult *Ncald*^KO/KO^ mice and suggest that NCALD has a specific role during postnatal brain maturation.

### NCALD Is Highly Elevated Postnatally and Regulates Neurogenesis in the Adult Mouse Brain

**Figure 3:**
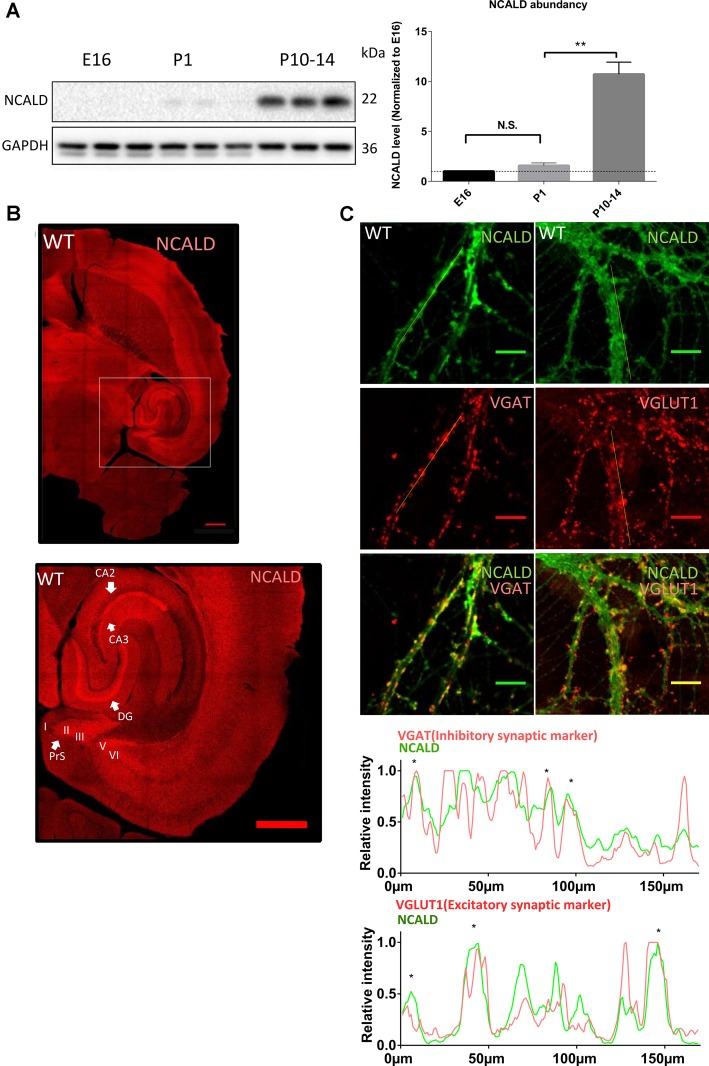
NCALD is enriched postnatally and in presynaptic terminals. **(A)** Western blot analysis of NCALD levels in brain lysates derived from the embryonic stage 16 (E16), postnatal day 1 (P1) and postnatal day 14 (P14) WT mice. Dots plots quantification reveals a 10-fold increase in the NCALD level in P10–14 brains; **P < 0.01; N.S. = not significant. **(B)** Representative confocal images of a WT brain immunostained for the NCALD, showing high protein expression in the forebrain and the midbrain and its abundance in the hippocampal and parahippocampal regions (magnified area); scale bar 500 μm. **(C)** Representative confocal images of cultured WT hippocampal neurons stained with NCALD antibody (green) and co-stained with synaptic markers VGLUT1 and VGAT (red), showing NCALD enrichment in the presynaptic terminals; scale bar 10 μm. Dotted white lines indicate areas taken for line plot analysis, where fluorescent signal for each channel is plotted relative to the distance. Asterisks in line plots represent the colocalization of NCALD with either VGLUT or VGAT. Uncropped Western blots are included in Supplementary Data Sheet 8.

To address the role of NCALD in brain maturation, we first analyzed NCALD levels in the WT mouse brain at various developmental time points (E16, P1 and P10–P14). We found that NCALD is present at very low levels during the embryonic stages and increases dramatically at P10–P14 (Figure 3A and Supplementary Figure S1D). In the adult brain, NCALD was found to be present throughout the brain, but was particularly enriched at multiple sites including hippocampal regions, dentate gyrus (DG) and CA3 as well as in the presubiculum (PreS; Figure 3B). Immunostaining of *Ncald*^KO/KO^ brain with NCALD antibody failed to show any signal (data not shown), demonstrating the specificity of the antibody. In cultured hippocampal neurons NCALD was co-localized with the excitatory vesicular glutamate transporter 1 (VGLUT1) and inhibitory vesicular GABA transporter (VGAT) presynaptic markers, indicating the presence of NCALD at presynaptic terminals (Figure 3C).

**Figure 4:**
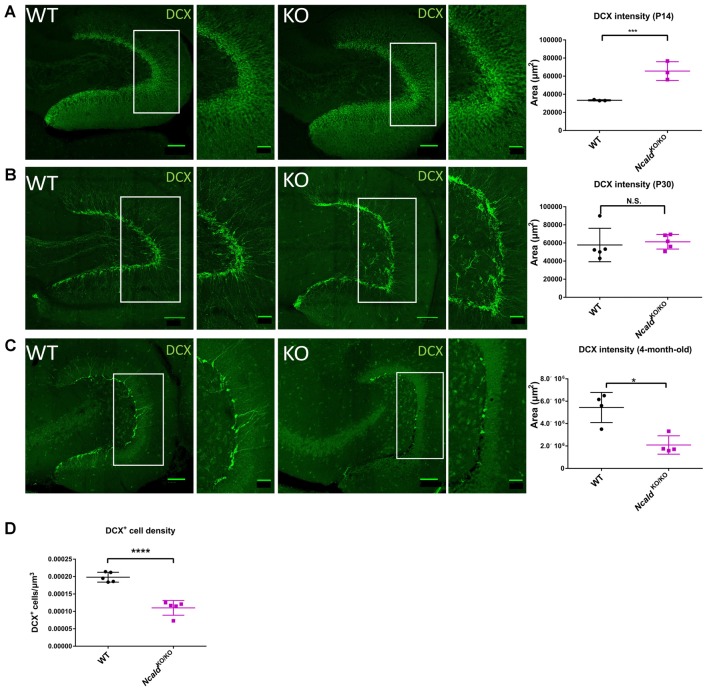
Disturbed adult neurogenesis in the hippocampus of *Ncald*^KO/KO^ mice.** (A)** Immunofluorescent analysis of doublecortin (DCX) positive neurons in the DG of P14 WT and *Ncald*^KO/KO^ animals; scale bars 100 μm and 40 μm (magnified insert); *N* = 3; ***P < 0.005. **(B)** Immunofluorescent analysis of DCX^+^ neurons in the DG of WT and *Ncald*^KO/KO^ animals at P30; scale bars 100 μm and 40 μm (magnified insert); *N* = 5; N.S. = not significant. **(C)** Immunofluorescent analysis of DCX^+^ neurons in the DG of 4-month-old WT and *Ncald*^KO/KO^ animals. DCX intensity is significantly lower in mice deficient for NCLAD; scale bar 100 μm and 40 μm (magnified inset); *N* = 5; *P < 0.05. **(D)** Dot plot analysis indicating a significant decrease in the DCX^+^ cell density in 4-month-old *Ncald*^KO/KO^ animals compared to WT controls; *N* = 5; ****P < 0.0001.

Since NCALD is strongly enriched in the hippocampus (Figure 3B) and homozygous *Ncald* knockout brains reveal defects in the architecture of the DG (Figure 2), we speculated that NCALD might be specifically involved in the regulation of the hippocampal morphology in the mouse brain. Since, defects in the hippocampal morphology in the adult *Ncald*^KO/KO^ animals can be associated with impaired postnatal neurogenesis, we first examined the overall cell proliferation in the DG using Ki-67, a protein only expressed in actively dividing cells (Scholzen and Gerdes, 2000), and found no general defect in cell proliferation (Supplementary Figure S5B). Following this, we analyzed newly generated neurons in the DG of *Ncald*^KO/KO^ animals and their control littermates by using Doublecortin (DCX) antibody, which labels newly generated granule cells undergoing migration (Couillard-Despres et al., 2005). We observed that the DCX intensity was significantly increased in *Ncald*^KO/KO^ mice at P14 whereas it was unaltered by homozygous *Ncald* deletion in the DG of P30 animals (Figures 4A,B). In contrast, in the DG of adult *Ncald*^KO/KO^ animals, we observed a significant reduction of DCX intensity as well as DCX^+^ neuronal density when compared to WT littermates (Figures 4C,D). Moreover, those few DCX^+^ neurons, which were present in the *Ncald*^KO/KO^ DG showed a tangential orientation instead of integration in the granule cell layer pointing towards a possible migration defect (Figure 4C).

To investigate this possibility, we analyzed the cortical layering in the 4-month-old *Ncald*^KO/KO^ animals. Improper organization of six cortical layers might be a consequence of the defects in neuronal migration, originating prenatally or early postnatally (Molyneaux et al., 2007). Immunohistochemical analysis of superficial and deep cortical layers stained with T-Box, Brain 1 (TBR1) and Cut Like Homeobox 1 (CUX1) antibodies revealed no alteration of cortical structure in *Ncald*^KO/KO^ animals (Supplementary Figures S4A,B), which suggests that NCALD does not regulate cortical layering. Taking into consideration a significant loss of DCX^+^ neurons in the adult *Ncald*^KO/KO^ animals, we also analyzed the heterozygous *Ncald*^KO/WT^ brains. We found that the DCX^+^ neuronal density as well as the DCX intensity was not significantly altered in *Ncald*^KO/WT^ brains, indicating that heterozygous *Ncald* knockout does not affect adult neurogenesis (Supplementary Figure S4C). Considering the possibility of degeneration of the DCX^+^ neurons, we immunostained *Ncald*^KO/KO^ brains for cleaved caspase 3 (an apoptotic marker), but we did not detect any apoptotic cell deaths in neurons lacking NCALD (data not shown).

To further investigate at which stage of adult neurogenesis NCALD regulates the DG granule cell function, we first quantified all the proliferating cells in the DG using the Ki-67 antibody as a cell-proliferating marker (Supplementary Figure S5A). However, we did not find any significant difference in the number of proliferating cells in adult *Ncald*^KO/KO^ compared to WT mice (Supplementary Figure S5B). Next, to label neural stem cells type 1 and type 2 we used GFAP and Nestin antibodies in combination with Ki-67, while type 3 neuronal stem cells were identified as being positive for Ki-67 along with DCX (Supplementary Figure S5A, and Sibbe et al., 2015). However, we did not observe any significant differences in the percentages of type1, 2 or 3 population of proliferating neuroblasts in *Ncald*^KO/KO^ compared to WT mice (Supplementary Figures S5C,D). Taken together, these data indicate that NCALD is not involved in neuroblast proliferation and likely regulates the DG granule cell function at the stage of neuronal maturation.

### NCALD Regulates Myelination in the Mouse Brain

Myelination is another important postnatal developmental event in the mouse brain (O’Rourke et al., 2014). Therefore, we next examined the myelination upon NCALD depletion by analyzing the levels of MBP in the 1-month-old *Ncald*^KO/KO^ animals. By using both immunohistochemistry and Western blotting, we found that MBP levels were significantly lower in brains lacking NCALD (Supplementary Figure S6A). Since the defects in the myelination could originate from a direct role of NCALD in oligodendrocytes, we analyzed the NCALD expression levels in oligodendrocytes by immunostaining with anti-APC antibody, an oligodendrocyte-specific marker (Lang et al., 2013). Co-localization analysis showed that NCALD is absent from these cells (Supplementary Figure S6B), indicating that NCALD does not function directly in oligodendrocytes.

To analyze if MBP reduction observed at P30 is a result of delayed myelination, we quantified the levels of MBP also at P14 and adult brains of *Ncald*^KO/KO^ mice (Supplementary Figures S6C–E). Indeed, we found no significant reduction in the MBP levels in adult brains, pointing towards a possible myelination delay in the 1-month-old *Ncald*^KO/KO^ animals. Since, it is known that the decreased myelination can be a consequence of reduced number of axons, we also analyzed the levels of neurofilament, an axonal marker in *Ncald*^KO/KO^ brain (Supplementary Figures S6C–E). We found no significant difference in the neurofilament levels ruling out decreased number of axons as the cause for the myelination defect observed in the *Ncald*^KO/KO^ brain.

### NCALD Regulates JNK Pathway

**Figure 5:**
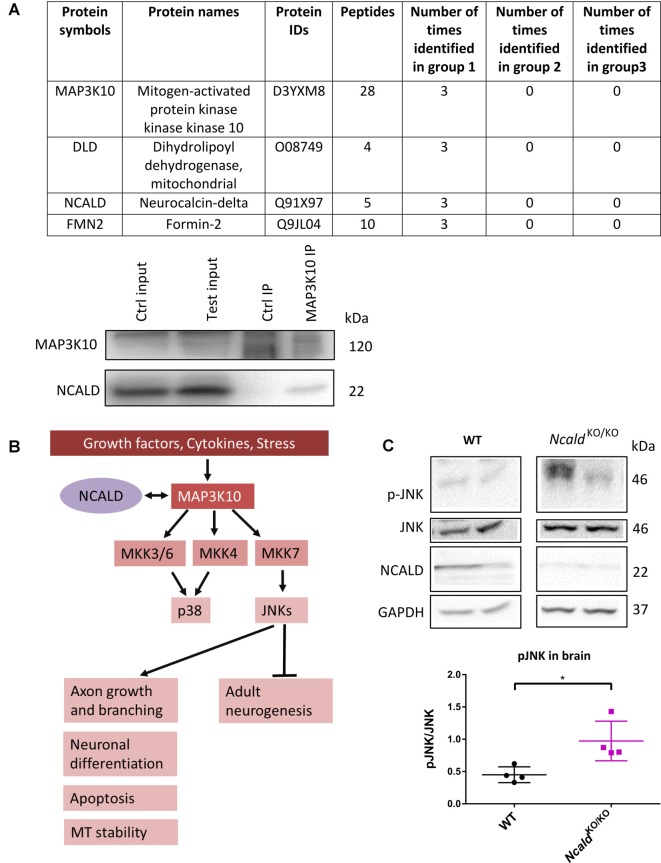
MAP3K10 interacts with NCALD and the downstream JUN N-terminal kinase (JNK) pathway is misregulated in *Ncald*^KO/KO^ brains. **(A)** Proteins identified to be present in all WT but not in *Ncald^KO/KO^* or negative control (beads only) brain lysates at P30. NCALD was immunoprecipitated using an NCALD-specific antibody and peptides were identified by mass spectrometry. Interaction between MAP3K10 and NCALD was confirmed by co-immunoprecipitation analysis. **(B)** Schematic illustration of MAP3K10-dependent regulation of JNK and P38 signaling pathways (modified from Hirai et al., 1997). **(C)** Representative Western blots and dot plots analysis showing a significant increase in JNK signaling in 4-month-old *Ncald*^KO/KO^ brain lysates compared WT controls. For quantification, pJNK levels were normalized to the total JNK levels; *N* = 4; *P < 0.05. Uncropped Western blots are included in Supplementary Data Sheet 8.

In order to unveil the mechanism underlying the morphological defects observed in the brain upon *Ncald* knockout, the interactome of NCALD was investigated using mass spectrometry (LC-MS) analysis of WT and *Ncald*^KO/KO^ brain samples at P30. Three different IPs were performed in triplicates with the following groups: (1) three IPs with NCALD antibody using WT brain lysate; (2) three IPs with NCALD antibody using *Ncald*^KO/KO^ brain lysates; and (3) three independent negative IPs (beats only) with WT lysates. The mass spectrometry result confirmed the absence of NCALD in the brain of *Ncald* knockout samples. The final list of identified proteins was based on the fact that the candidate was present only in the WT NCALD IPs (group 1) but absent from all IPs of groups 2 and 3. Only three proteins met these criteria (Figure 5A). Due to the highest number of identified peptides for MAP3K10 this protein was selected for further analysis. We first confirmed the interaction of MAP3K10 and NCALD in the brain by co-immunoprecipitation using the MAP3K10 antibody (Figure 5A).

MAP3K10 functions as a part of the MAP kinase pathway, upstream of two major MAPKs, namely JNK and P38 (Hirai et al., 1997). JNK is known to regulate various cellular processes, including apoptosis, neuronal differentiation, axonal growth and branching. Additionally, JNK has recently been reported to regulate the adult neurogenesis (Coffey, 2014; Mohammad et al., 2018; Figure 5B). Taking into account the impaired adult neurogenesis in *Ncald*^KO/KO^ animals, we evaluated the JNK and pJNK level in adult NCALD-depleted brains by Western blotting. We found that in *Ncald*^KO/KO^ brains the phosphorylation of JNK (pThr 183 and pTyr 185) was significantly upregulated compared to wildtype littermates (Figure 5C). At the same time, the phosphorylation of JNK in heterozygous *Ncald* knockout brains was not altered (Supplementary Figure S7A). These data suggest a specific role for NCALD in the MAP3K10-regulated JNK activation. These findings, together with the fact that alterations in the JNK pathway have already been reported in SMA (Genabai et al., 2015), prompted us to subject the spinal cord lysates from *Ncald*^KO/KO^ and WT animals to LC/MS analysis. Our results from the spinal cord confirmed the data obtained from the brain and revealed the MAP3K10 as one of the top interaction partners of NCALD (data not shown). However, we did not find any significant differences in p-JNK in the spinal cord from *Ncald*^KO/KO^ animals, suggesting a brain-confined modulation of JNK pathway by NCALD (Supplementary Figure S7B).

### Heterozygous and Homozygous Ncald Knockout Increases Axonal Length

**Figure 6:**
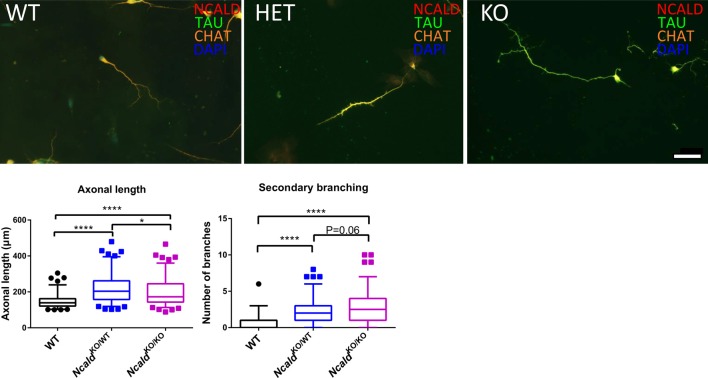
Heterozygous and homozygous *Ncald* deletion results in longer axons in spinal motor neurons (MNs). Cultured MNs isolated from WT, *Ncald*^KO/WT^ and *Ncald*^KO/KO^ E13.5 mouse embryos were stained with NCALD, TAU and Choline acetyltransferase (CHAT) antibodies. Nuclei were labeled with DAPI; scale bar 50 μm. Dot plot analysis reveals a statistically significant increase in the average axon length and secondary axonal branching in *Ncald*^KO/WT^ and *Ncald*^KO/KO^ neurons compared to WT; *N* = 108, 104, 105; ****P < 0.0001; *P < 0.05. Twenty-five to Seventy-five percent values covered by each box plot, line represents median and dotted outliers at <5% and >95% CI.

Finally, since NCALD reduction has been shown to act protective in MNs derived from the spinal cord of SMA transgenic mice (Riessland et al., 2017), we analysed the effect of NCALD deficiency on MN morphology in *Ncald*^KO/WT^ and *Ncald*^KO/KO^ mice. We examined the morphology of cultured MNs with a MN specific marker ChAT as well as microtubule stabilising protein TAU. In accordance with our previous findings (Riessland et al., 2017), we found that both 50% reduction and a complete deletion of NCALD significantly increased the length of MN axons compared to controls (Figure 6). Interestingly, *Ncald*^KO/KO^ MNs had significantly shorter axons when compared to MNs isolated from *Ncald*^KO/WT^ mice. Since this phenotype in *Ncald*^KO/KO^ mice was accompanied by a strong, but not significant increase in a number of secondary branches, we reason that decreased axonal length in *Ncald*^KO/KO^ neurons results from their increased secondary branching (Figure 6). On other hand, we found that in *Ncald*^KO/WT^ and *Ncald*^KO/KO^ hippocampal neurons axonal length was not altered (Supplementary Figure S7C).

## Discussion

The main findings of this study are: (1) NCALD is especially abundant in certain regions of the hippocampus like DG and CA3 as well as in the PreS; (2) homozygous loss of *Ncald* impairs hippocampal morphology with reduction in subgranuler zone length and causes enlargement of lateral ventricles in 4-month-old mice; (3) NCALD levels increase dramatically during the early postnatal stages between P10 and P14; (4) adult *Ncald*^KO/KO^ animals reveal severe changes in the adult neurogenesis in the DG; (5) NCALD interacts with MAP3K10 and regulates the JNK pathway in the brain; and (6) none of the deleterious phenotypes found in homozygous *Ncald* knockout mice are observed in heterozygous *Ncald* knockout animals, while NCALD reduction is sufficient to increase the axonal length of MN; this indicates that half reduction of NCALD is safe to be used as a potential SMN-independent therapy for SMA patients.

### Physiological Significance of NCALD in Postnatal Brain Development and Adult Neurogenesis

We found that NCALD levels are steadily increasing at postnatal stages until P14. Moreover, we found that homozygous *Ncald* knockout significantly increases the intensity of DCX (a marker for newly differentiated and immature cells) in the early adolescent (P14) brain. These data are in agreement with our previous finding, where NCALD reduction in cultured MN-like cells has been shown to promote their early differentiation (Riessland et al., 2017). In contrast to the P14, we found that the loss of NCALD in the adult DG significantly decreases the DCX intensity as well as the DCX^+^ cell number. The transition of immature DCX positive cells to mature granule cells is most pronounced during P7–P28, hence it is a crucial stage for the formation of the SGZ (Radic et al., 2017). Furthermore, the full electrophysiological maturation of new granule cells progresses over the period of ca. 3–7 weeks after cell division (Overstreet-Wadiche and Westbrook, 2006; Zhao et al., 2006). Therefore, potential effects of NCALD loss on brain morphology would arise earliest at 7 week-of age. This is strongly in line with our observations, where no significant changes in the hippocampus could be detected in P30 animals, however they were prominent in adult *Ncald* knockout mice. Additionally, we found that NCALD depletion does not affect the proliferation of various types of neuroblasts in the adult DG. However, future studies using the BrdU incorporation method are required to fine-tune the onset of these aberrations and reinforce our findings. Moreover, it has been shown that adult neurogenesis can directly regulate the volume and morphology of the hippocampus (Fuss et al., 2014; Baptista and Andrade, 2018). Therefore, the loss of adult neurogenesis observed in adult *Ncald* knockout animals could be an accumulated loss of DCX^+^ immature neurons, which were not able to integrate into the granule layer. Interestingly, in a recent database of RNA expression profiles during adult neurogenesis, *Ncald* expression has been shown to specifically increase at the immature granule cell stage (Hochgerner et al., 2018). These data corroborate our finding that adult *Ncald*^KO/KO^ animals show a significant reduction specifically in immature granule cell population of neurons (Figures 4C,D) and points towards a stage specific role of NCALD in the adult neurogenesis.

We show that the changes in adult neurogenesis observed in *Ncald*^KO/KO^ mice are accompanied by hyperactivation of the JNK pathway in the brain of NCALD depleted animals. These data are in agreement with the recent study showing that JNK acts as a negative regulator of adult neurogenesis (Mohammad et al., 2018). Considering that the gradient regulation of JNK pathway during the embryonic development of the brain has been implicated in the migration and maturation of neurons (Hirai et al., 2002), JNK activation could well be the mechanism responsible for the aberrant migration of newborn neurons in adult *Ncald*^KO/KO^ animals. Therefore, further rescue studies of adult neurogenesis defects in *Ncald*^KO/KO^ animals treated with blood-brain barrier permeable JNK inhibitor (Mohammad et al., 2018) as well as by targeted overexpression of *Ncald* can deepen our understanding of NCALD function.

### Implications of NCALD in Other Physiological Functions

Most of the NCALD-enriched brain regions described in the current study have already been reported (Girard et al., 2015), except for the PreS. PreS is a part of the parahippocampal region (Witter et al., 2000) which has been strongly implicated in spatial navigation (Boccara et al., 2010). Thus, we suggest that spatial navigation might be disturbed by NCALD loss in the adult mice, however this hypothesis needs to be tested in future experiments.

Although NCALD has been previously implicated in synaptic function (Kedracka-Krok et al., 2015), our study provides the first direct evidence of NCALD localization at synaptic boutons of hippocampal neurons *via* colocalization of NCALD with VGLUT1, an excitatory synaptic marker and VGAT, an inhibitory synaptic marker.

### NCALD in Neurodevelopmental Diseases

Multiple studies indicate the importance of hippocampal shape and morphology in cognitive functions (Smith et al., 2012; Voineskos et al., 2015). Smaller hippocampi, as well as severe morphological disturbances in hippocampal shape observed in *Ncald*^KO/KO^ animals implicate NCALD function in cognition. Indeed, NCALD has been associated with schizophrenia (Vercauteren et al., 2007) and autism (Ben-David et al., 2011), both representing neurodevelopmental disorders associated with cognitive loss and characterized by enlargement of lateral ventricles both in human patients (Movsas et al., 2013) and genetic mouse models (Pletnikov et al., 2008). A similar ventricle enlargement phenotype, as well as impairment in postnatal development has been observed for *Ncald*^KO/KO^ mice in the current study. Moreover, phenotypic data available at the International Mouse Phenotyping Consortium platform indicate that *Ncald*^KO/KO^ mice are hyperactive and anxious and exhibit severe reduction of body mass,^1^ similar to what we observed in our *Ncald*^KO/KO^ animals. Henceforth, a detailed study of NCALD function in the pathophysiology of schizophrenia, autism, depression, stress-related disorders like bipolar disorder and anxiety, as well as a comprehensive analysis cognitive behavior of *Ncald*^KO/KO^ animals have a potential to reveal a disease mouse model for neurodevelopmental disorders with subtle behavioral symptoms, thereby improving our understanding of such disorders.

### Can NCALD Reduction be Used for Future SMN-Independent SMA Therapy?

SMA therapeutics reached a very important milestone in the last 2 years, with the FDA and EMA approval of Nusinersen for treatment of SMA patients outside the clinical trial (Scoto et al., 2017). Nusinersen is a modified ASO which binds to the *SMN2* pre-mRNA at an intronic splice silencer site, whereby it disrupts the interaction with negative splicing factors. This in turn promotes the inclusion of exon 7 in the *SMN2* mRNA thereby enhancing the SMN protein levels (Rigo et al., 2014). Nusinersen has shown significant benefits in the progress of SMA patients, however it could not fully revert the normal motor functions in patients (Talbot and Tizzano, 2017). Specifically, in case of type I SMA patients, who usually have only two *SMN2* copy and some even only one (Feldkötter et al., 2002), increasing the SMN level *via* one or two *SMN2* copies may not be sufficient to fully counteract SMA pathology. Therefore, there is a need for SMN-independent therapies, which can work in combination with Nusinersen or other SMN-dependent therapies (Finkel et al., 2017; Mendell et al., 2017).

We have already published the therapeutic importance of NCALD reduction in the context of SMA (Riessland et al., 2017). Supporting our previous studies here, we show that reduction of NCALD even by 50%, such as in *Ncald*^KO/WT^ mice has no systemic consequences for the animal physiology. Furthermore, we found that NCALD reduction in *Ncald*^KO/WT^ MNs increases the axonal length independent of SMA.

### JNK Pathway in SMA

We observed that *Ncald*^KO/KO^ can alter JNK phosphorylation in the brain but not in the spinal cord (Figure 5C and Supplementary Figure S7B). Although both the spinal cord and the brain are the part of the CNS, there are significant variations in their metabolic, functional and defense mechanisms (Panov et al., 2011). These variations could underlie the neuronal type-specific response of JNK signaling pathway to the NCALD depletion in either brain or spinal cord. Moreover, we found that while *Ncald*^KO/KO^ significantly upregulated the JNK phosphorylation (Figure 5C), half reduction (*Ncald*^KO/WT^) has no effect on the JNK activation (Supplementary Figure S7A), suggesting its safe use in SMA therapy.

Interestingly, increased JNK phosphorylation has also been detected in the spinal cord of SMA mice, which however is linked to reduced SMN levels and cellular stress response in spinal cord MNs (Genabai et al., 2015). Consequently, inhibition of JNK in SMA mice by using the D-JNKI1 inhibitor ameliorated the SMA pathology (Schellino et al., 2018). We speculate that different signaling pathways operate to regulate JNK signaling in SMA disease mouse model and in *Ncald* knockout condition. In this scenario, cellular stress response causes JNK activation in SMA mice, whereas *Ncald* knockout leads to the loss of MAP3K10–NCALD interaction, which causes the increased JNK phosphorylation in the brain.

Taken together, our study identifies a novel link between NCALD and adult neurogenesis in the hippocampus, possibly *via* MAP3K10-JNK pathway and establishes the safety of NCALD reduction as viable option for a combinatorial therapy to treat SMA.

## Author Contributions

AU, BW and NK designed the project and wrote the manuscript and co-authors read and confirmed the finally submitted manuscript. AU carried out all experiments with the help of SH, SS, AK, LT-B, NM-F, MO, RR, VG, MK and NK. SH carried out the mass spectrometry and proteomics analysis. BW and NK supervised the project.

## Conflict of Interest Statement

The authors declare that the research was conducted in the absence of any commercial or financial relationships that could be construed as a potential conflict of interest.

## Footnotes

^1^http://www.mousephenotype.org/
