# Supplementary material for: Neurocalcin Delta Knockout Impairs Adult Neurogenesis Whereas Half Reduction Is Not Pathological
Source: Front Mol Neurosci. 2019 Feb 12;12:19. doi: 10.3389/fnmol.2019.00019 (PMC6396726; doi:10.3389/fnmol.2019.00019)
Supplement: Supplementary file 1 [file Data_Sheet_1.PDF]

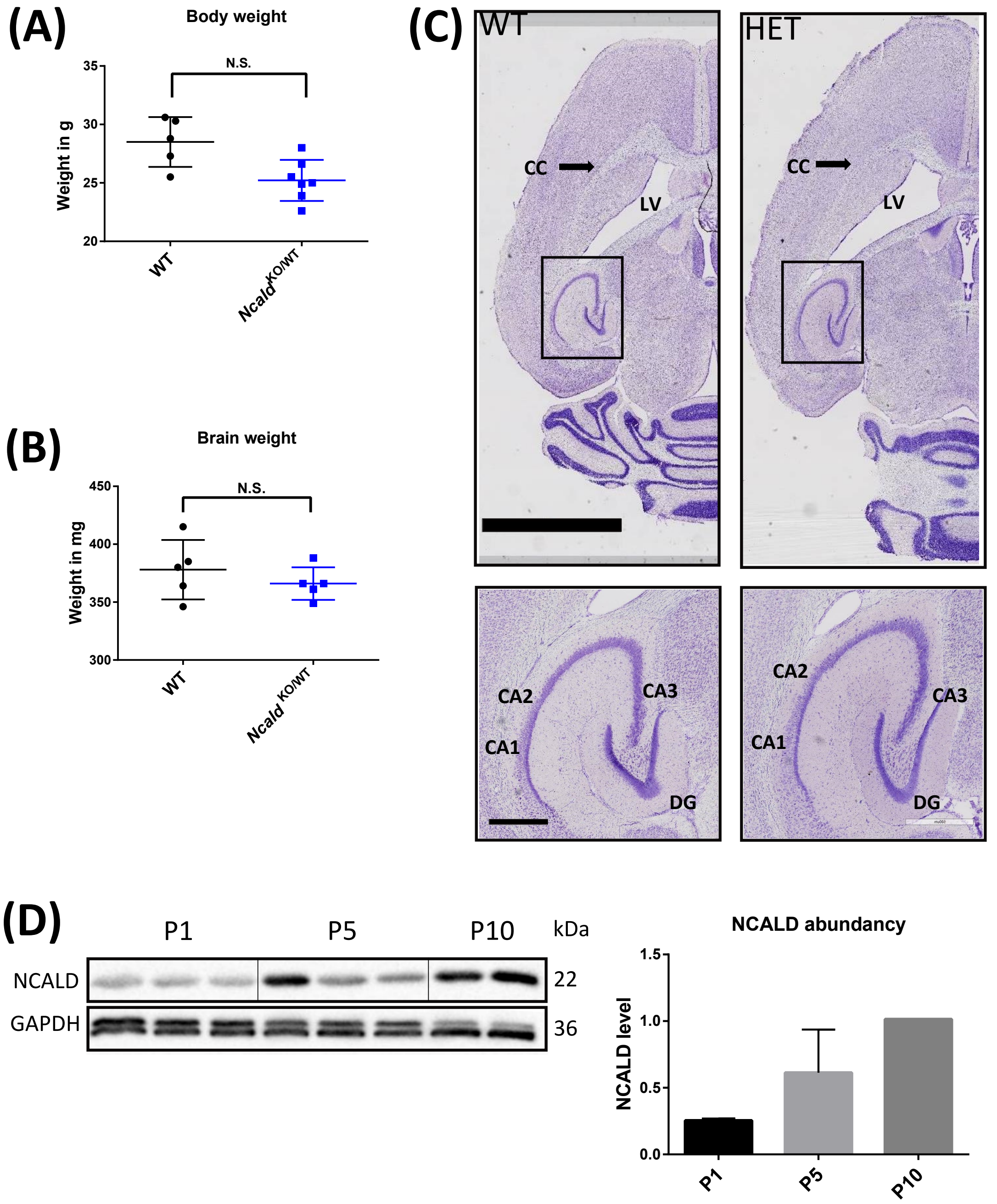

Supplementary figure 1. Unaltered body weight and brain morphology in mice heterozygous for *Ncald*. (A) Dot plot analysis revealing unaltered body weight of 4-month-old *Ncald*$^{KO/WT}$ compared to WT animals; N=7 and N=5, respectively, N.S. = not significant. **(B)** Dot plot analysis revealing unaltered brain weight of *Ncald*$^{KO/WT}$ and WT animals; N= 5. N.S. = not significant **(C)** Nissl-stained brain sections from 4-month-old *Ncald*$^{KO/WT}$ animals reveal unaltered brain morphology in *Ncald*$^{KO/WT}$ compared to WT littermates. N=4; scale bar 3 mm and 500 µm (magnified inset). **(D)** Western blot analysis shows a gradual increase in NCALD levels from P1 to P10. Graph representing the quantification of the Western blot. Uncropped Western blots are included in Supplementary Data Sheet 8.
