# Supplementary material for: Neurocalcin Delta Knockout Impairs Adult Neurogenesis Whereas Half Reduction Is Not Pathological
Source: Front Mol Neurosci. 2019 Feb 12;12:19. doi: 10.3389/fnmol.2019.00019 (PMC6396726; doi:10.3389/fnmol.2019.00019)
Supplement: Supplementary file 2 [file Data_Sheet_2.PDF]

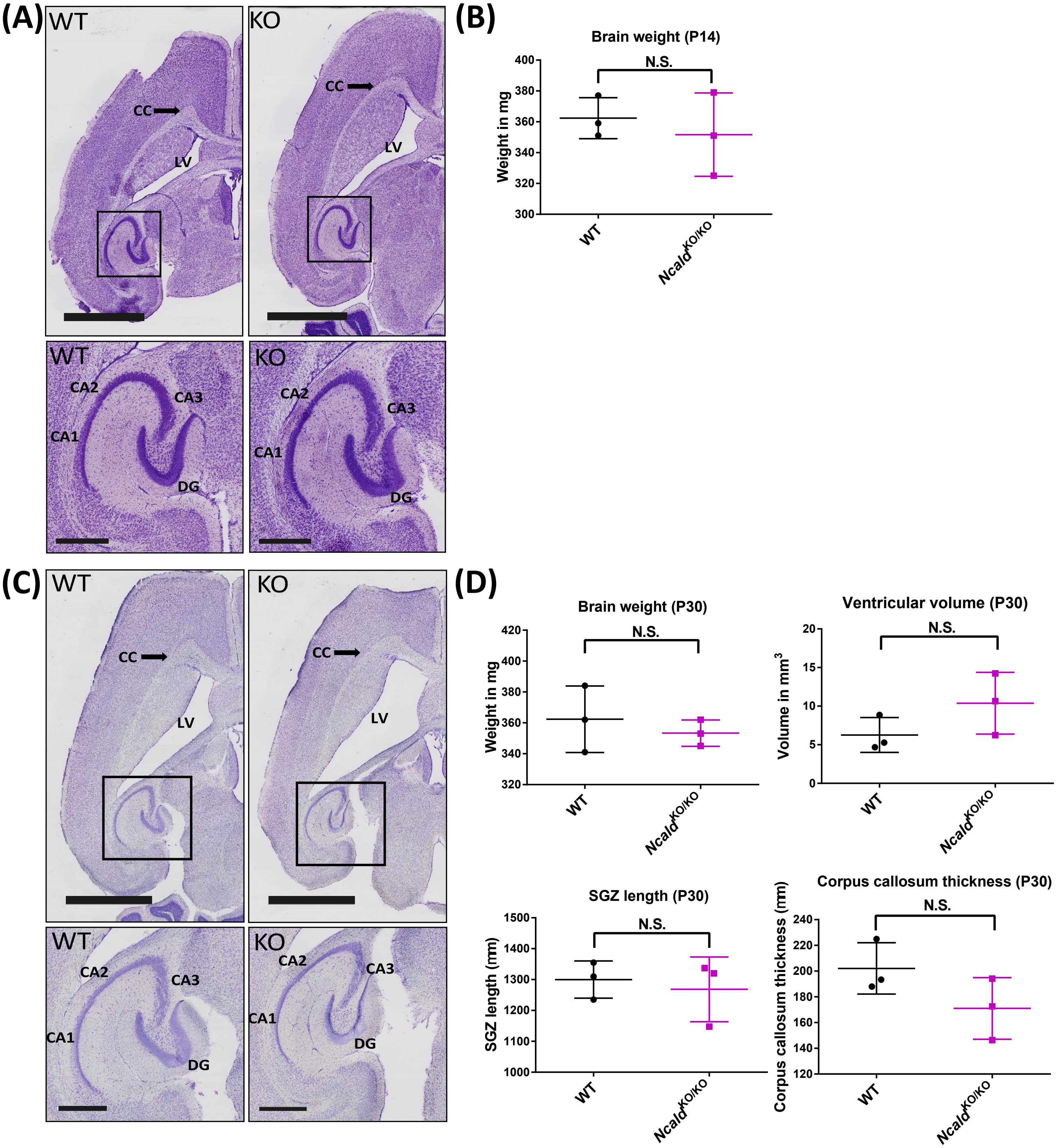

Supplementary figure 2. Unaltered gross morphology of *Ncald* KO/KO brains at P14 and P30.

(A) Nissl-stained brain sections from *Ncald*$^{KO/KO}$ mice at P14; scale bars 2 mm and 500 µm (magnified inset). **(B)** Dot plot analysis showing no significant difference in the body weight of *Ncald*$^{KO/KO}$ mice compared to WT controls at P14; N=3, N.S. = not significant. **(C)** Nissl-stained brain sections of *Ncald*$^{KO/KO}$ mice at P30; scale bars 2 mm and 500 µm (magnified inset). **(D)** Dot plot analysis showing no significant difference in the body weight, ventricular volume, the SGZ length and the corpus callosum thickness of *Ncald*$^{KO/KO}$ compared to WT animals at P30; N=3, N.S. = not significant.
