# Supplementary material for: Neurocalcin Delta Knockout Impairs Adult Neurogenesis Whereas Half Reduction Is Not Pathological
Source: Front Mol Neurosci. 2019 Feb 12;12:19. doi: 10.3389/fnmol.2019.00019 (PMC6396726; doi:10.3389/fnmol.2019.00019)
Supplement: Supplementary file 3 [file Data_Sheet_3.PDF]

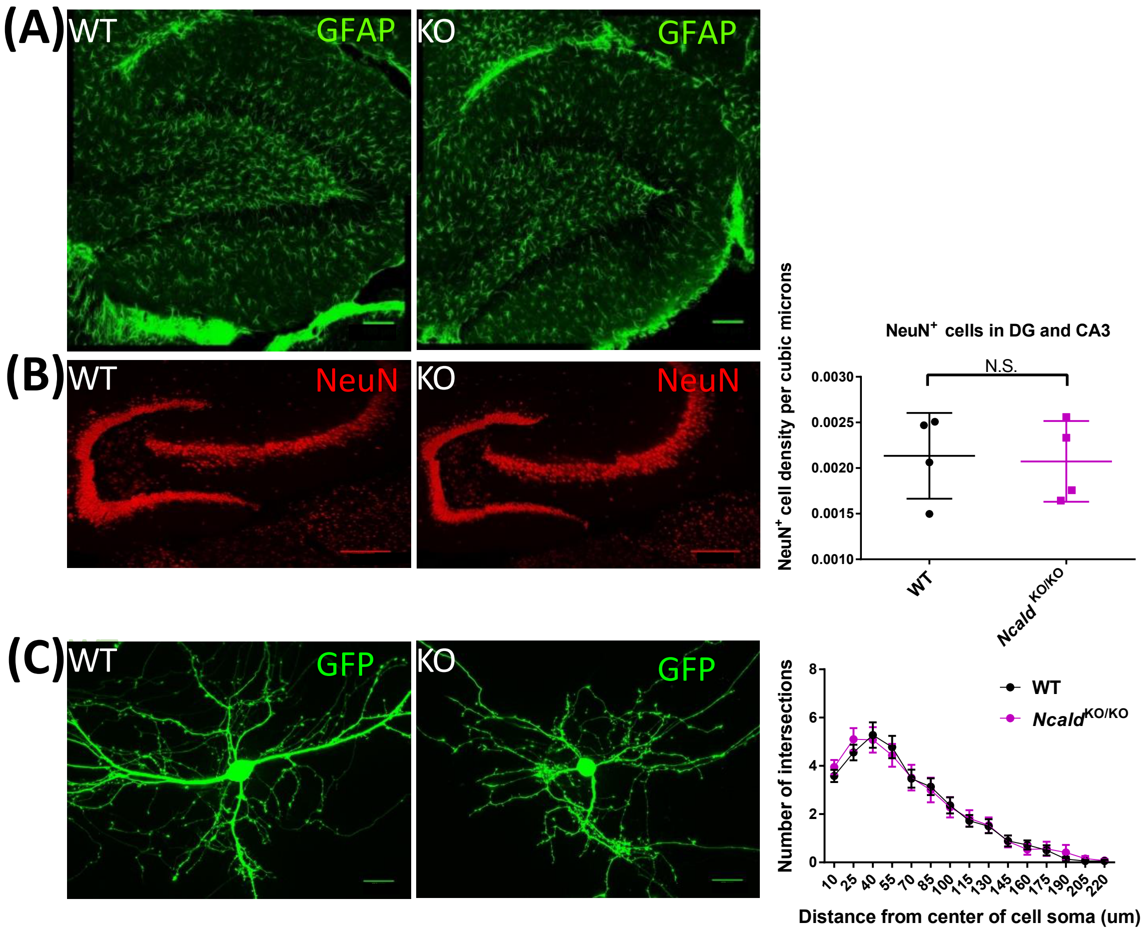

Supplementary figure 3. Cell type specific and morphological analysis of the *Ncald*KO/KO brains. (A) Representative confocal images of 4-month-oldand *Ncald*KO/KO brains immunostained for glial fibrillary acidic protein (GFAP), a marker of astrogliosis; scale bar 100 μm. **(B)** Representative confocal images of 4-month-old WT and *Ncald*KO/KO brains immunostained for neuronal marker NeuN and dot plot analysis of neuronal cell density in the CA3 and the DG of adult WT and *Ncald*KO/KO brains; N=3; scale bar 200 μm. N.S. = not significant **(C)** GFP-transfected cultured hippocampal neurons from WT and *Ncald*KO/KO mice fixed at DIV 14 and the graph representing the Sholl analysis of dendritic neuronal complexity; N=36 and N=39, respectively; scale bar 20 μm.
