# Supplementary material for: Neurocalcin Delta Knockout Impairs Adult Neurogenesis Whereas Half Reduction Is Not Pathological
Source: Front Mol Neurosci. 2019 Feb 12;12:19. doi: 10.3389/fnmol.2019.00019 (PMC6396726; doi:10.3389/fnmol.2019.00019)
Supplement: Supplementary file 4 [file Data_Sheet_4.PDF]

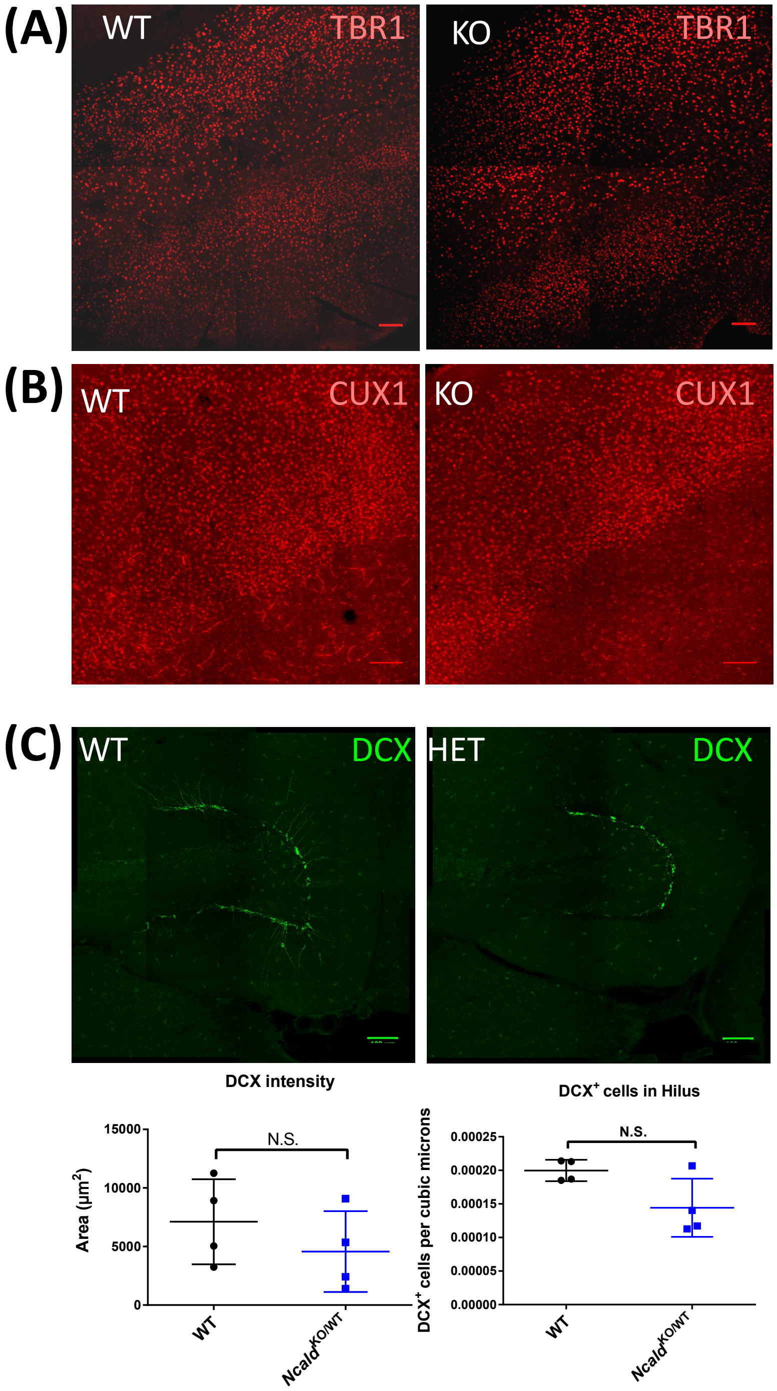

Supplementary figure 4. Cortical layer organization in *Ncald*$^{KO/KO}$ brains and adult hippocampal neurogenesis in *Ncald*$^{KO/WT}$ brains. (A) Representative confocal images of brain sections from 4-month-old WT and *Ncald*$^{KO/KO}$ brains immunostained for T-box brain 1 (TBR1), which show the correctly confined deep layer in the cortex of *Ncald*$^{KO/KO}$ mice; scale bar 100 µm. **(B)** Representative confocal images of brain sections from 4-month-old WT and *Ncald*$^{KO/KO}$ brains immunostained for Cut-like homeobox 1 (CUX1), which show the correctly confined superficial layer in the cortex of *Ncald*$^{KO/KO}$ mice; scale bar 100 µm. **(C)** Representative confocal images of brain sections from 4-month-old WT and *Ncald*$^{KO/WT}$ brains immunostained for DCX, which show unaltered DCX intensity and the number of DCX positive neurons in the DG of *Ncald*$^{KO/WT}$ brains; N=4; scale bar 100 µm, N.S. = not significant.
