# Supplementary material for: Neurocalcin Delta Knockout Impairs Adult Neurogenesis Whereas Half Reduction Is Not Pathological
Source: Front Mol Neurosci. 2019 Feb 12;12:19. doi: 10.3389/fnmol.2019.00019 (PMC6396726; doi:10.3389/fnmol.2019.00019)
Supplement: Supplementary file 5 [file Data_Sheet_5.PDF]

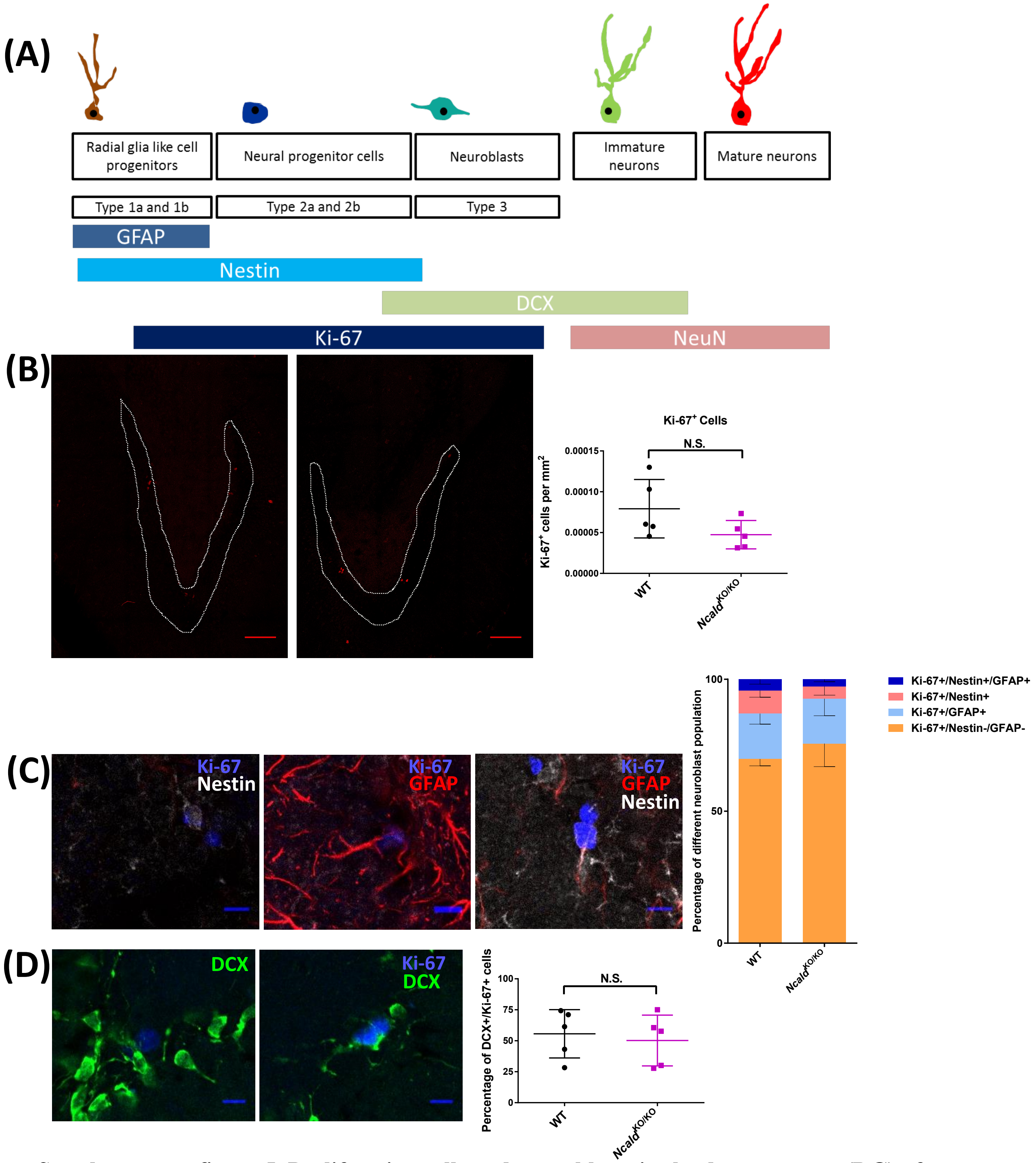

Supplementary figure 5. Proliferating cells and neuroblasts in the dentate gyrus (DG) of *Ncald*$^{\mathrm{KO/KO}}$ brains. (A) Scheme showing the different cell population and cell type specific markers during proliferation, differentiation and maturation of granule cells during adult neurogenesis **(B)** Representative confocal images of the DG from 4-month-old WT and *Ncald*$^{\mathrm{KO/KO}}$ mice immunostained for Ki-67 (proliferation specific marker) and dot plot analysis showing Ki-67 positive cell density; N=4; scale bar 100 µm. **(C)** Representative confocal images of the DG from 4-month-old WT and *Ncald*$^{\mathrm{KO/KO}}$ mice immunostained for Ki-67 and co-immunostained for Nestin and glial fibrillary acidic protein (GFAP). The graph represents the percentage of Ki-67 cells double-positive for either Nestin or GFAP, or triple-positive for both in

the DG of 4-month-old WT and *Ncald*$^{KO/KO}$ mice; N=5; scale bar 10 µm. **(D)** Representative confocal images of the DG from 4-month-old WT and *Ncald*$^{KO/KO}$ mice immunostained for Ki-67 and co-immunostained for DXC and quantification showing percentages of above-mentioned population in the WT and *Ncald*$^{KO/KO}$ animals; N=5; scale bar 10 µm, N.S. = not significant.
