# Supplementary material for: Neurocalcin Delta Knockout Impairs Adult Neurogenesis Whereas Half Reduction Is Not Pathological
Source: Front Mol Neurosci. 2019 Feb 12;12:19. doi: 10.3389/fnmol.2019.00019 (PMC6396726; doi:10.3389/fnmol.2019.00019)
Supplement: Supplementary file 6 [file Data_Sheet_6.PDF]

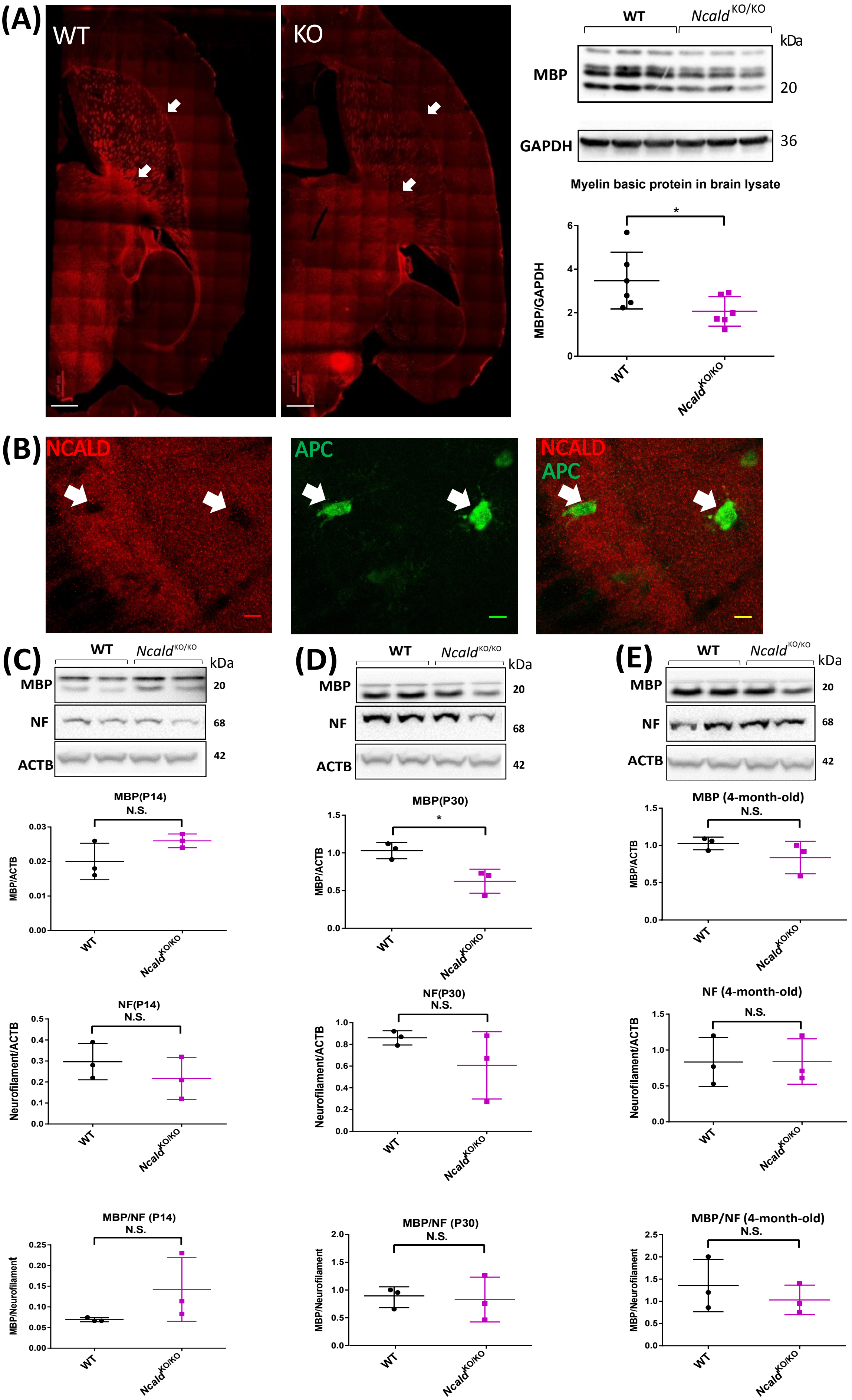

Supplementary figure 6. Decreased myelination in *Ncald*$^{KO/KO}$ brains. (A) Representative confocal images of 1-months-old WT and *Ncald*$^{KO/KO}$ brains immunostained for myelin basic protein (MBP); scale bar 500 µm. Representative Western blot and dot plot analysis of MBP levels in lysates derived from 1-month-old WT and *Ncald*$^{KO/KO}$ brains; N=6, * P< 0.05. **(B)** Co-

immunostaining of NCALD and adenomatosis polyposis coli (APC; oligodendrocyte specific marker) showing absence of NCALD in oligodendrocytes (P=0.25, Mander's coefficient) scale bar 10 μm. **(C-E)** Representative Western blots and dot plot analysis of MBP and neurofilament (NF, axonal specific marker) levels in lysates derived from WT and *Ncald*$^{KO/KO}$ brains from **(C)** P14; N=3, **(D)** P30; N=3; $P< 0.05$, and **(E)** 4-month-old animals; N=3.; * $P< 0.05$, N.S.= not significant. Uncropped Western blots are included in Supplementary Data Sheet 8.
