# Supplementary material for: Neurocalcin Delta Knockout Impairs Adult Neurogenesis Whereas Half Reduction Is Not Pathological
Source: Front Mol Neurosci. 2019 Feb 12;12:19. doi: 10.3389/fnmol.2019.00019 (PMC6396726; doi:10.3389/fnmol.2019.00019)
Supplement: Supplementary file 7 [file Data_Sheet_7.PDF]

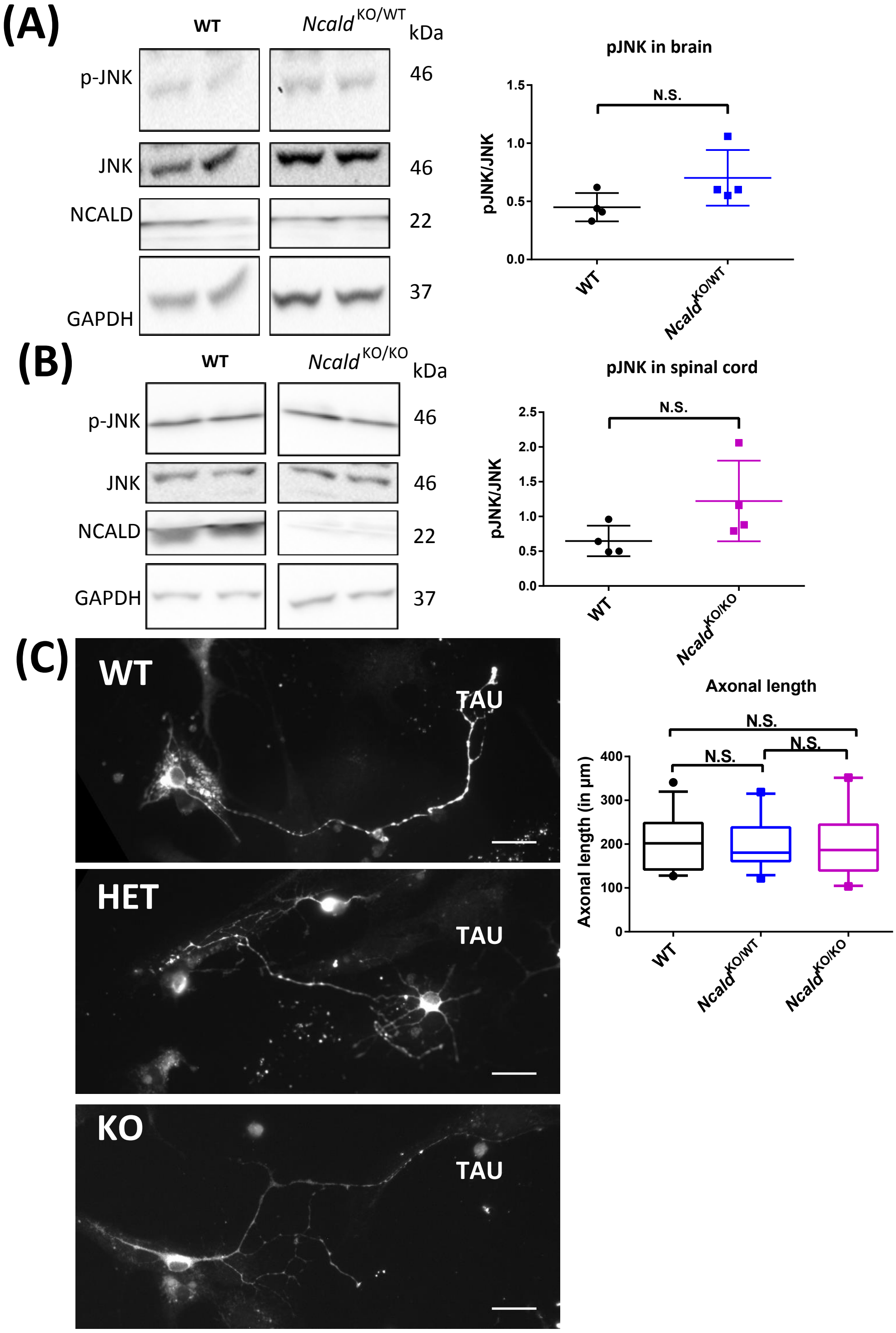

Supplementary figure 7. Analysis of pJNK signalling in *Ncald*$^{KO/WT}$ brains and *Ncald*$^{KO/KO}$ spinal cord. (A) Representative Western blots and dot plot analysis of pJNK (46 kDA) and total JNK levels in WT and *Ncald*$^{KO/WT}$ littermate brains; N=4, N.S. = not significant **B)** Representative Western blots and dot plot analysis of pJNK and total JNK levels in the spinal cord of 4-month-old WT versus *Ncald*$^{KO/KO}$ littermates; N=4, N.S. = not significant **(C)** Cultured hippocampal neurons isolated from WT, *Ncald*$^{KO/WT}$ and *Ncald*$^{KO/KO}$ P2 pups were immunostained with TAU antibody at DIV 5. ; scale bar 20 µm. Graph representing unaltered average axon length in *Ncald*$^{KO/WT}$ and *Ncald*$^{KO/KO}$ neurons compared to the WT; N= 25, 24, 27 (from 3 embryos). 25-75% values covered by each box plot, line represents median and dotted outliers at <5% and >95% CI, N.S. = not significant. Uncropped Western blots are included in Supplementary Data Sheet 8.
