# Supplementary material for: Neurocalcin Delta Knockout Impairs Adult Neurogenesis Whereas Half Reduction Is Not Pathological
Source: Front Mol Neurosci. 2019 Feb 12;12:19. doi: 10.3389/fnmol.2019.00019 (PMC6396726; doi:10.3389/fnmol.2019.00019)

Data sheet 8: Uncropped Western blots

Figure1 (A)

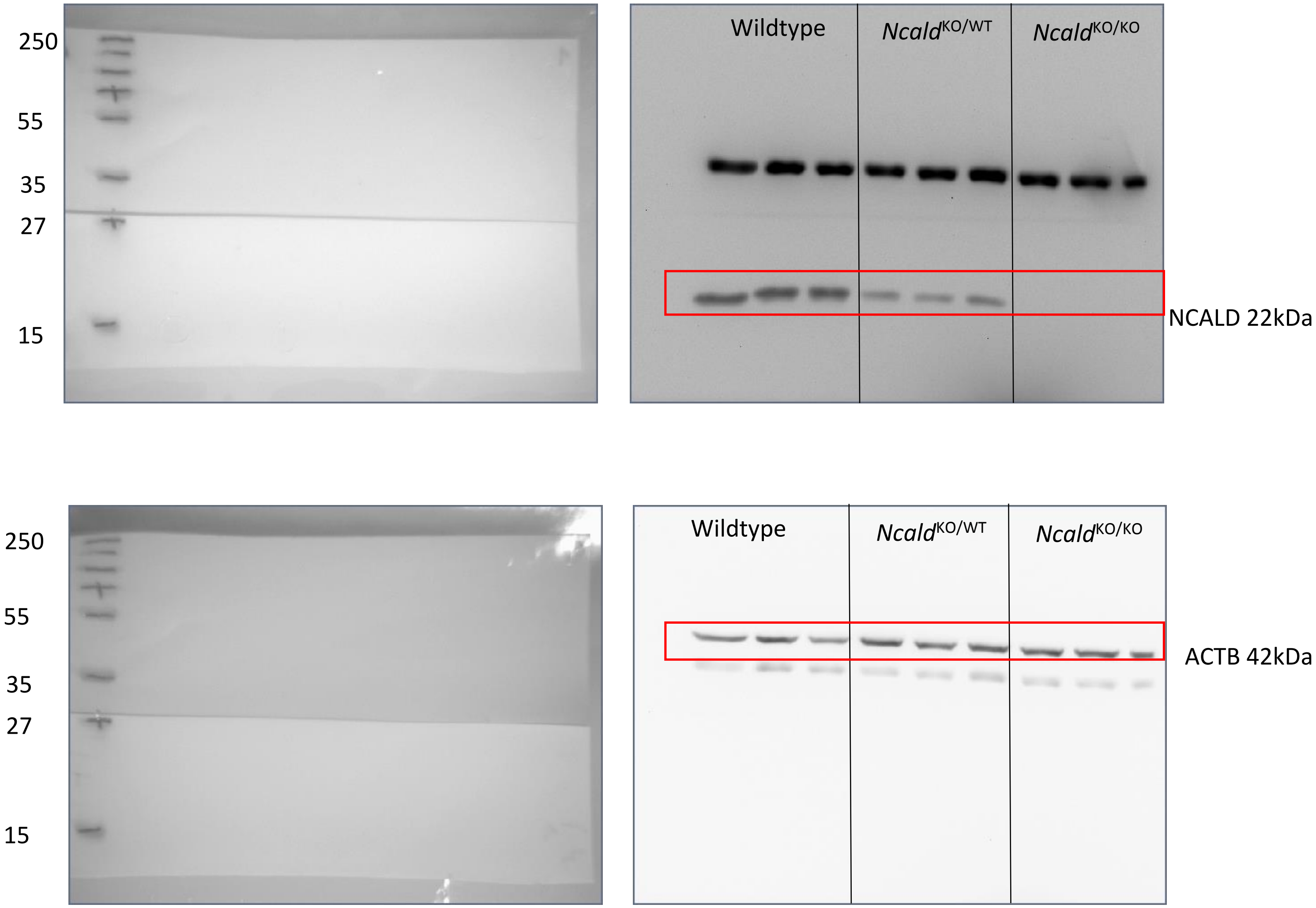

250
55
35
27
15
Wildtype
Ncald KO/WT
Ncald KO/KO
NCALD 22kDa
250
55
35
27
15
Wildtype
Ncald KO/WT
Ncald KO/KO
ACTB 42kDa

Figure3 (A)

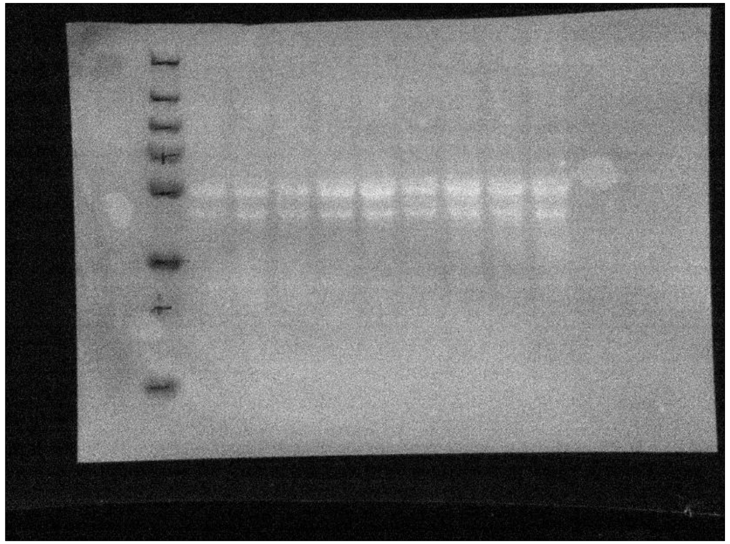

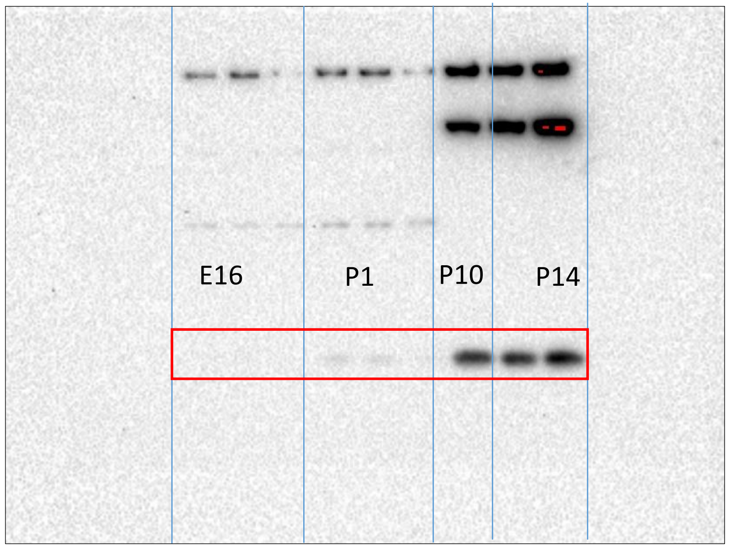

NCALD 22 kDa

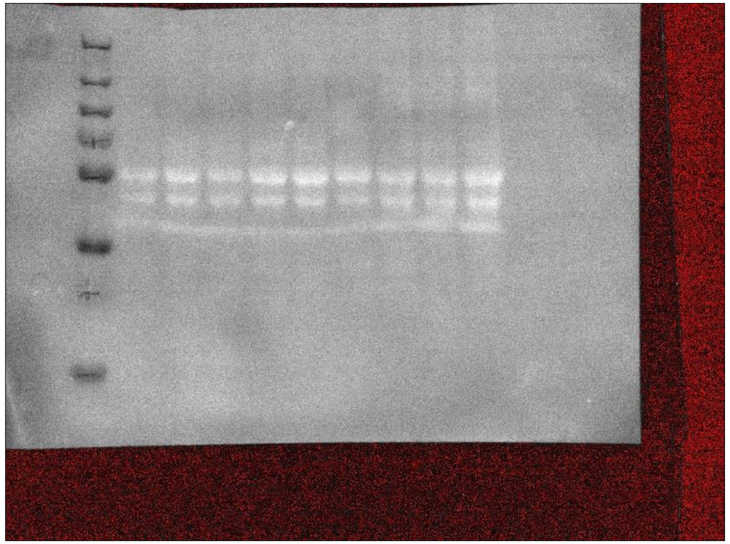

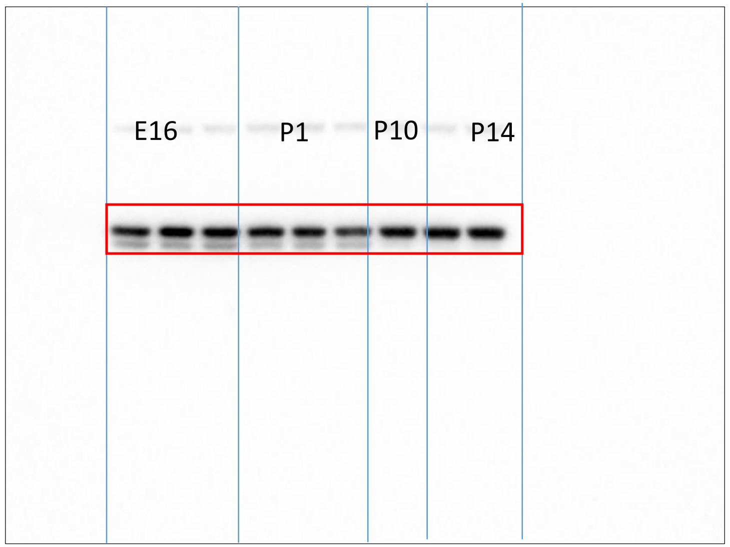

GAPDH 37 kDa

Figure5 (A)

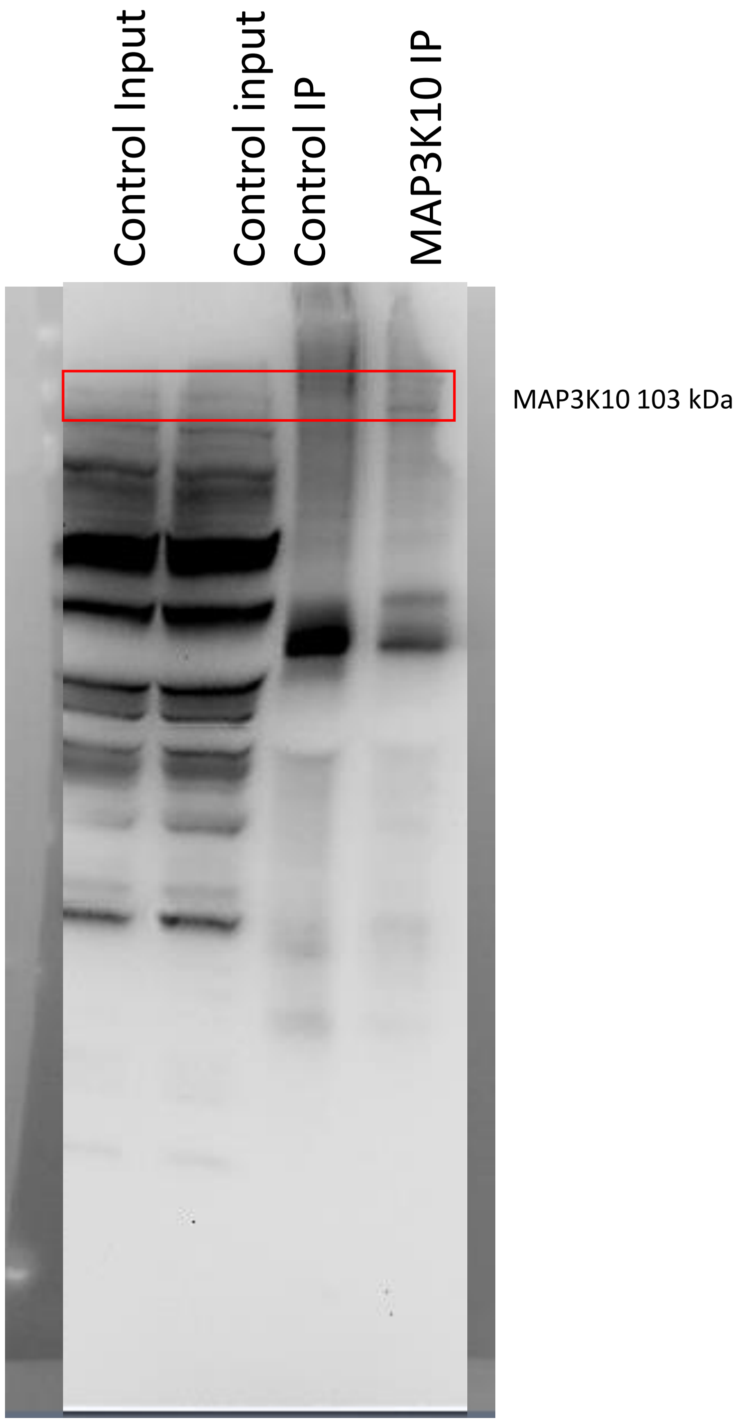

Control Input
Control input
Control IP
MAP3K10 IP
MAP3K10 103 kDa

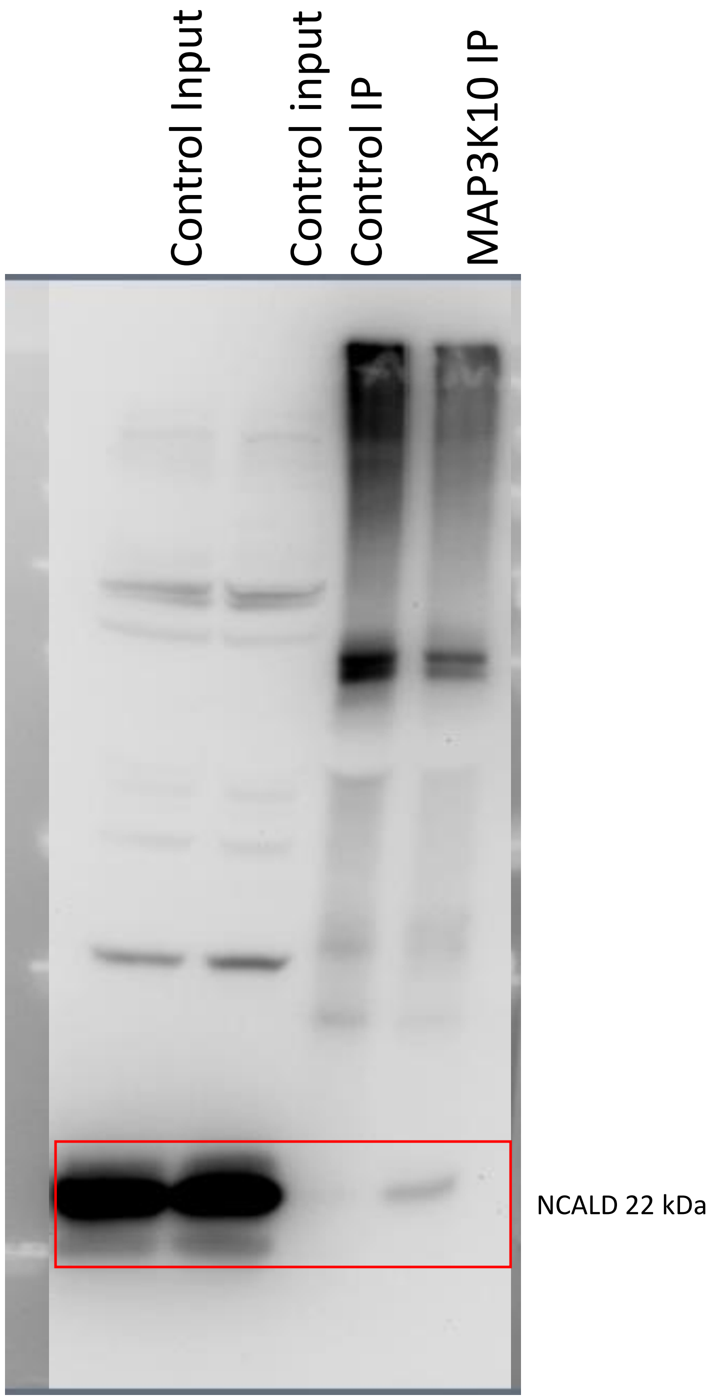

Control Input
Control input
Control IP
MAP3K10 IP
NCALD 22 kDa

Figure5 (C)

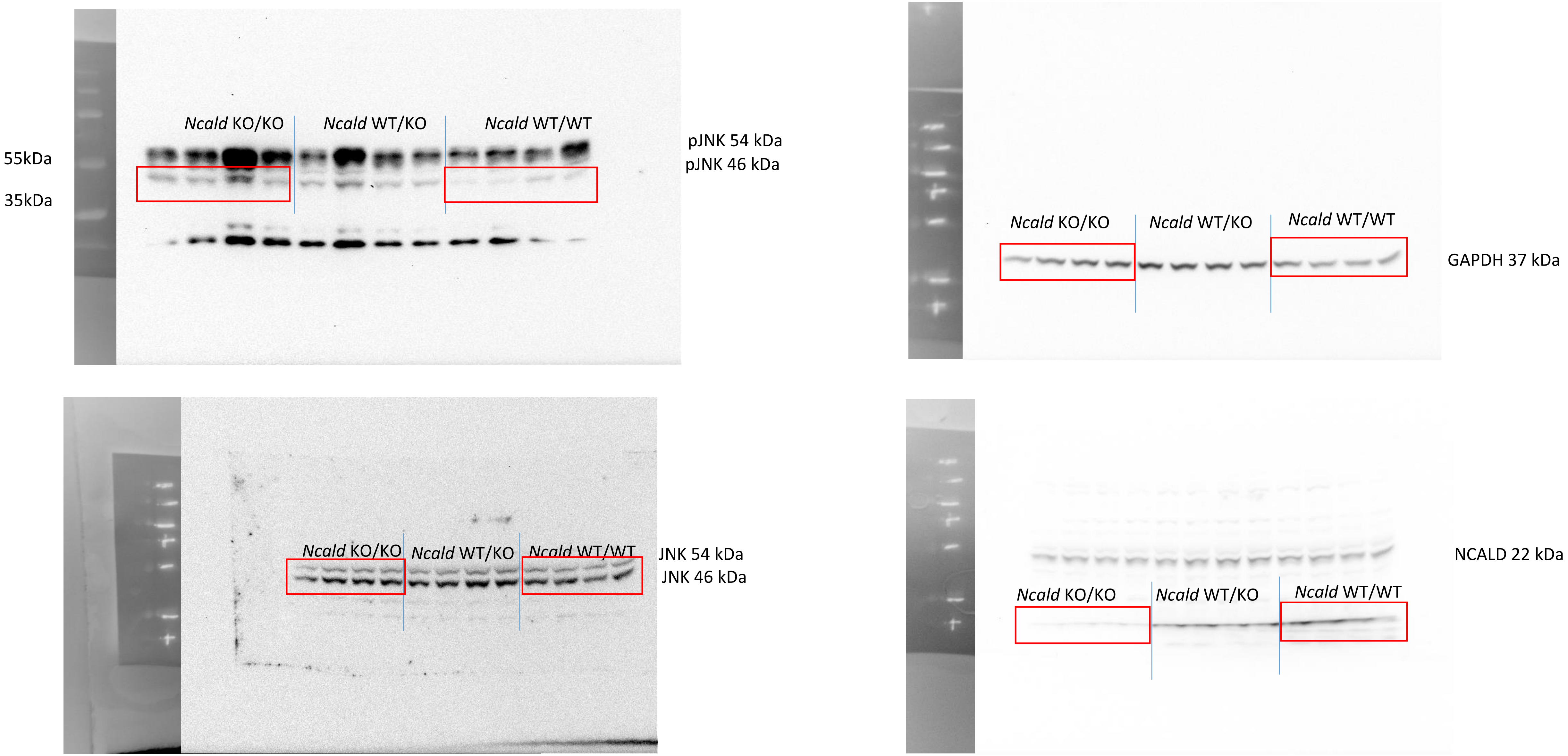
Ncald KO/KO
Ncald WT/KO
Ncald WT/WT
55kDa
35kDa
pJNK 54 kDa
pJNK 46 kDa
Ncald KO/KO
Ncald WT/KO
Ncald WT/WT
GAPDH 37 kDa
Ncald KO/KO
Ncald WT/KO
Ncald WT/WT
JNK 54 kDa
JNK 46 kDa
NCALD 22 kDa
Ncald KO/KO
Ncald WT/KO
Ncald WT/WT

Supplementary figure 1 (D)

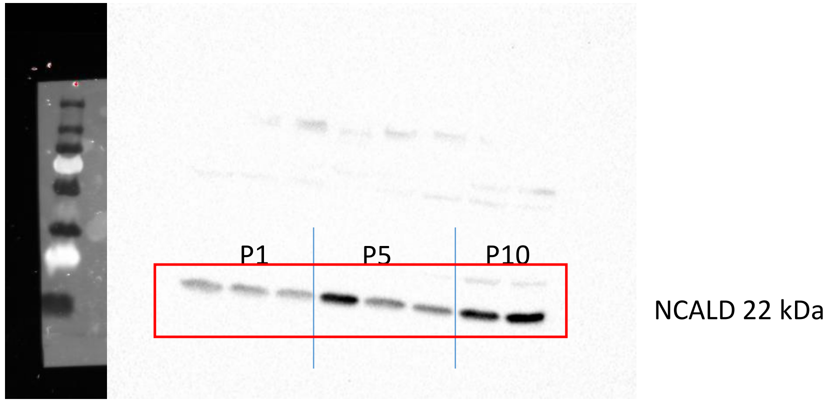

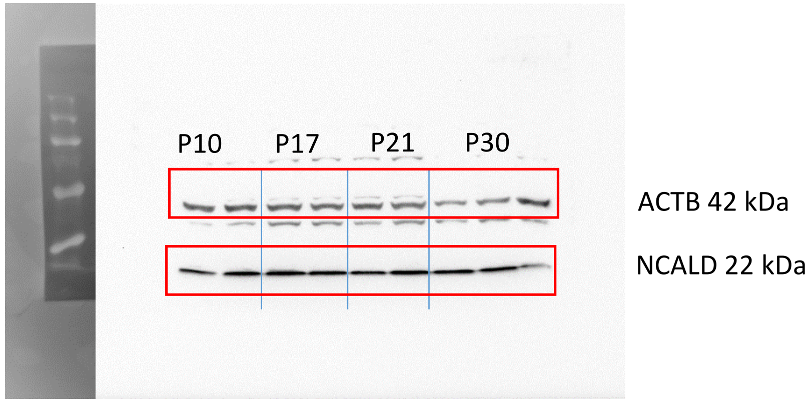

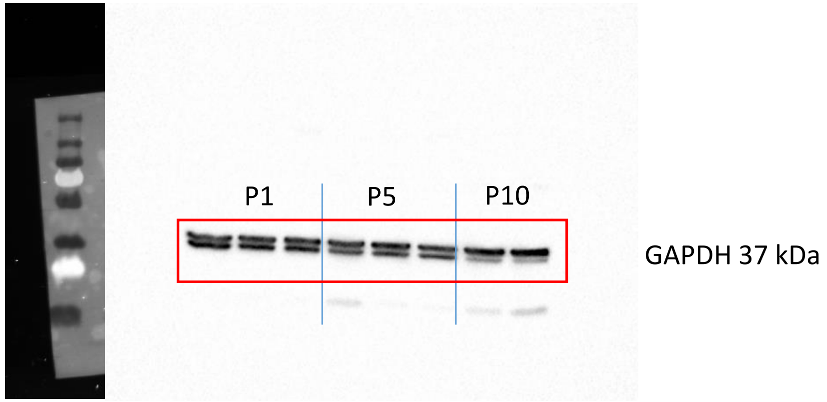

Supplementary figure 6 (A)

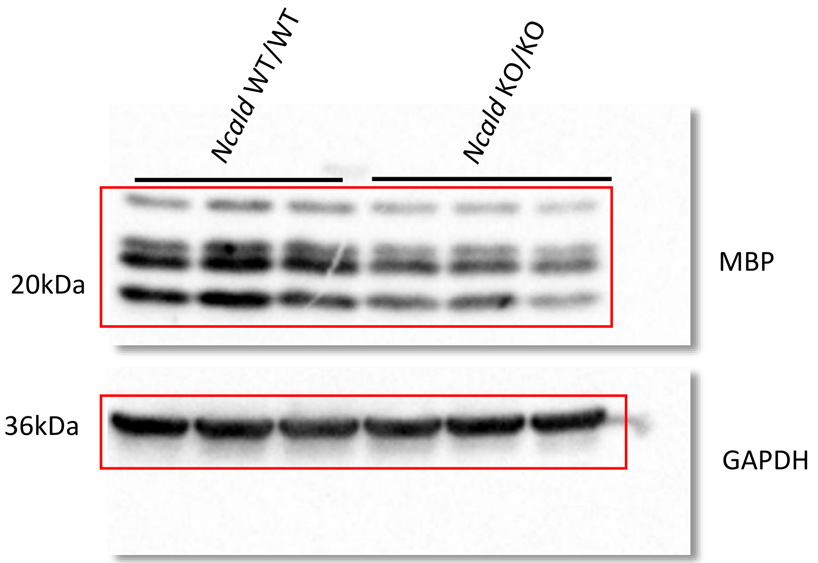

Supplementary figure 6 (C)

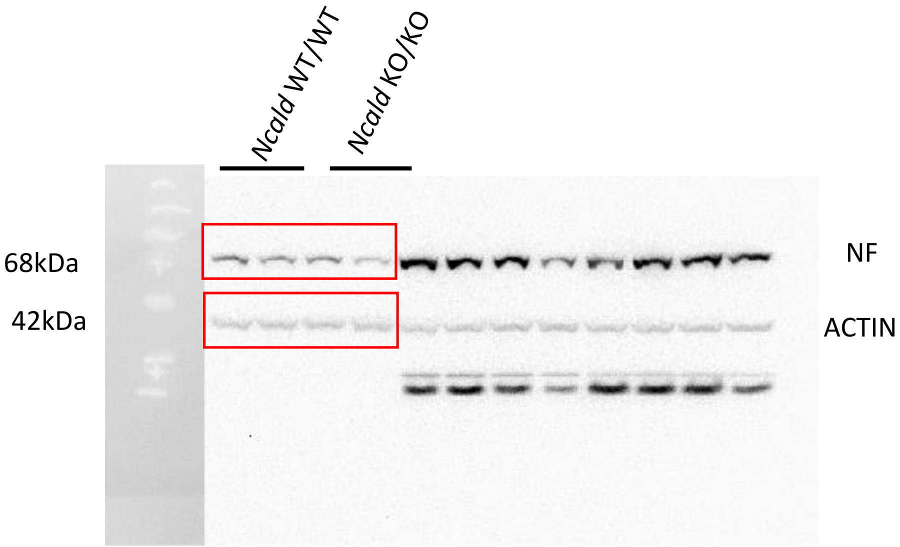

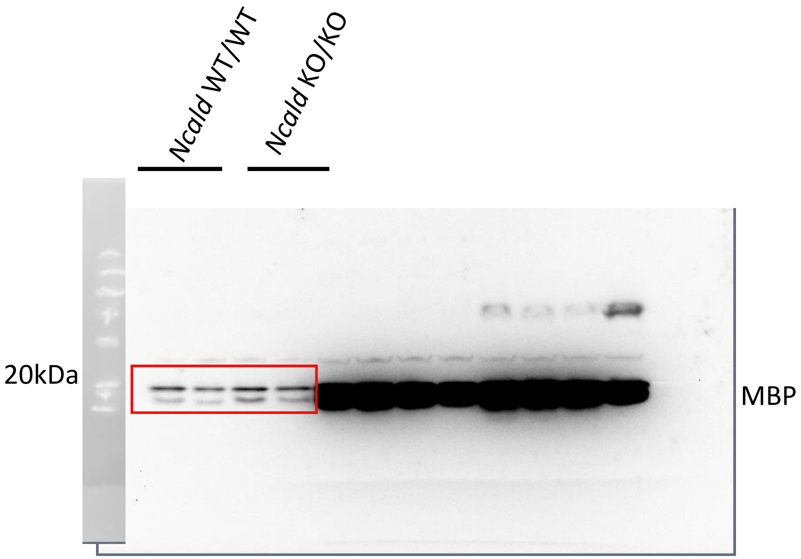

Supplementary figure 6 (D)

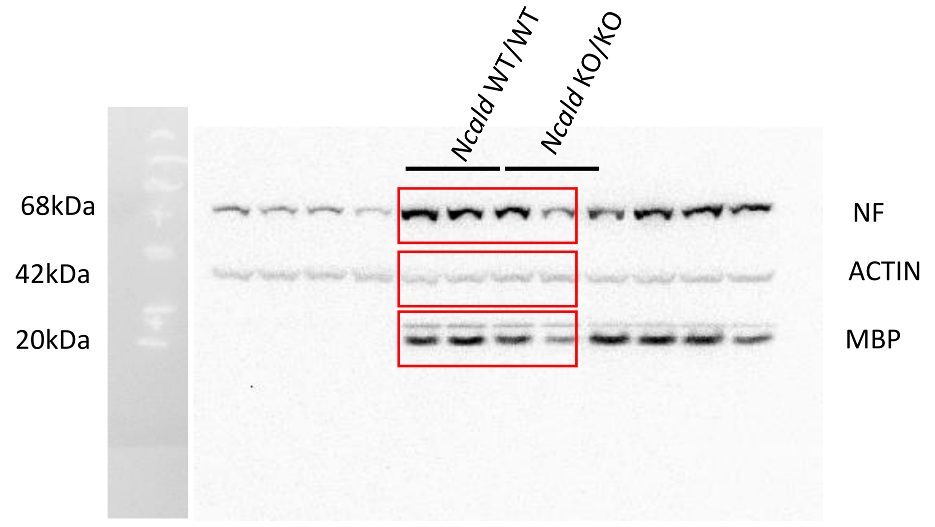

Supplementary figure 6 (E)

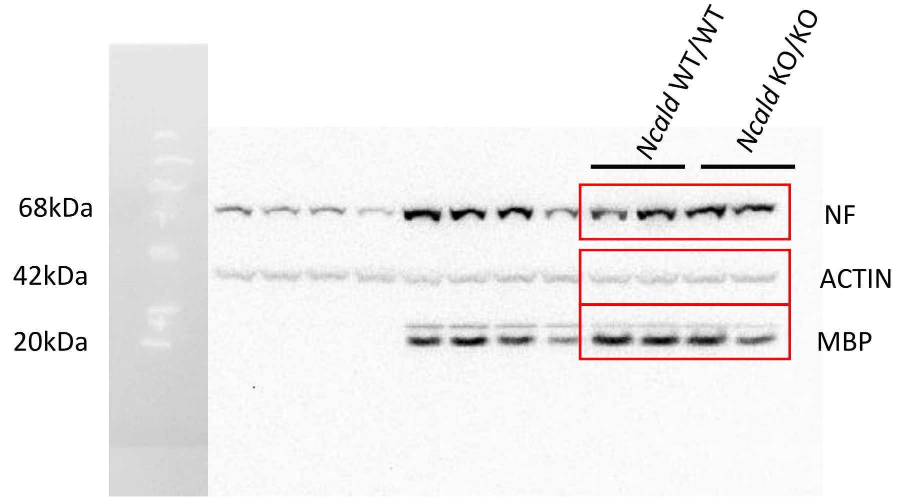

Supplementary figure 7(A)

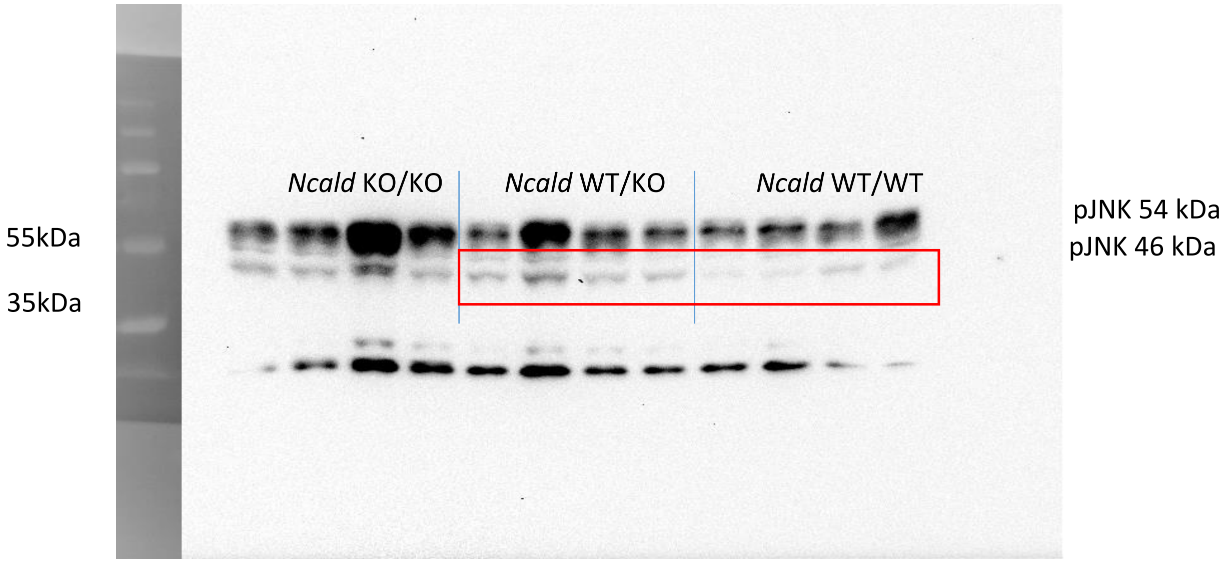

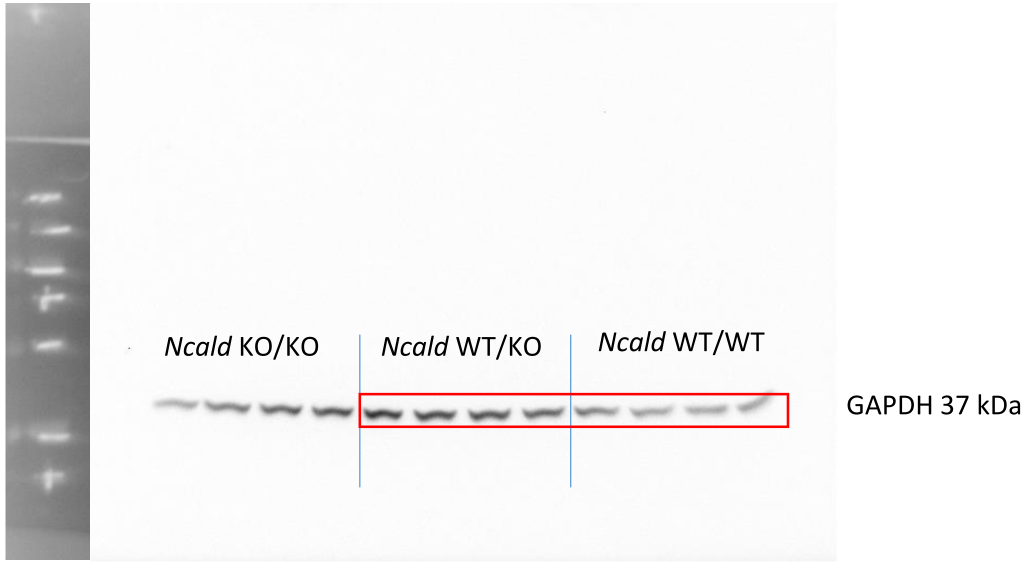

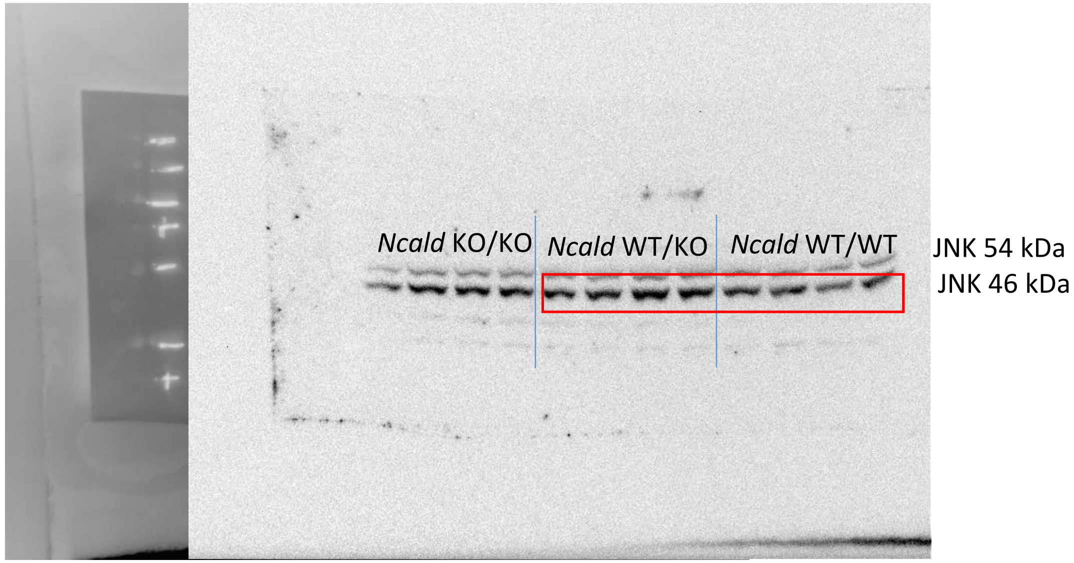

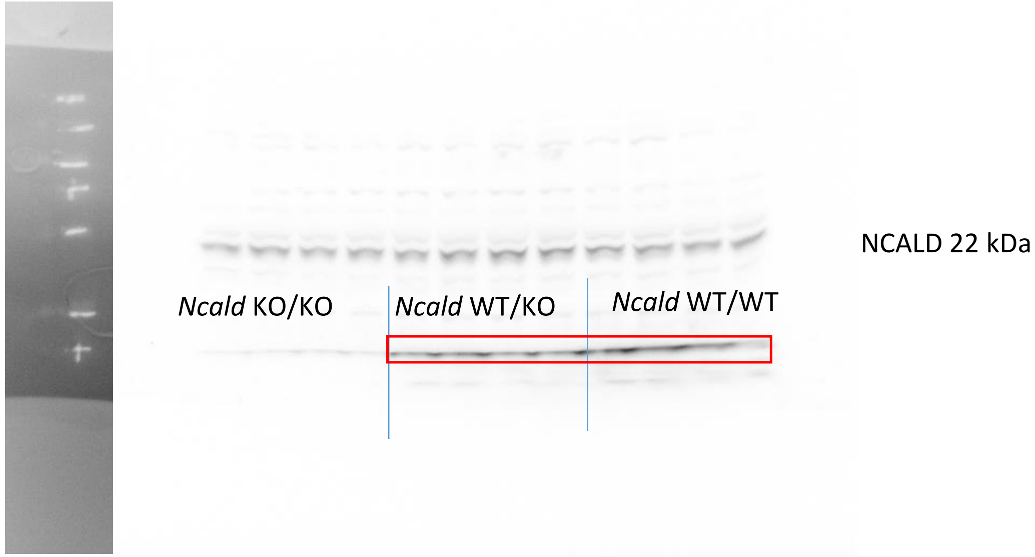

Supplementary figure 7 (B)

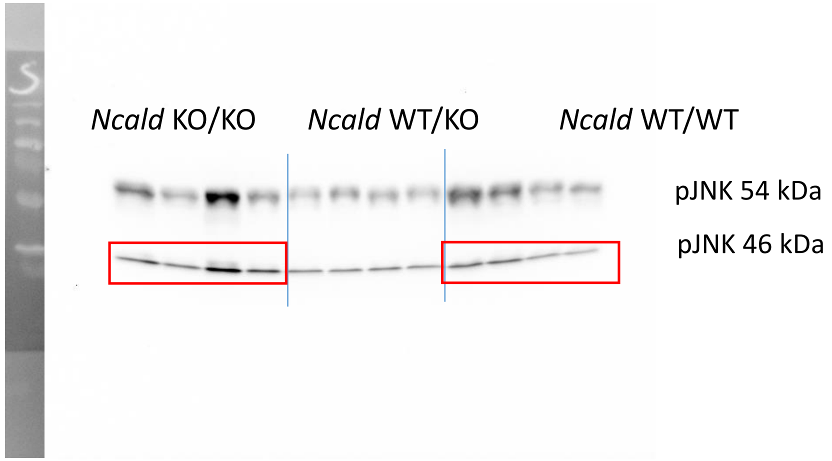

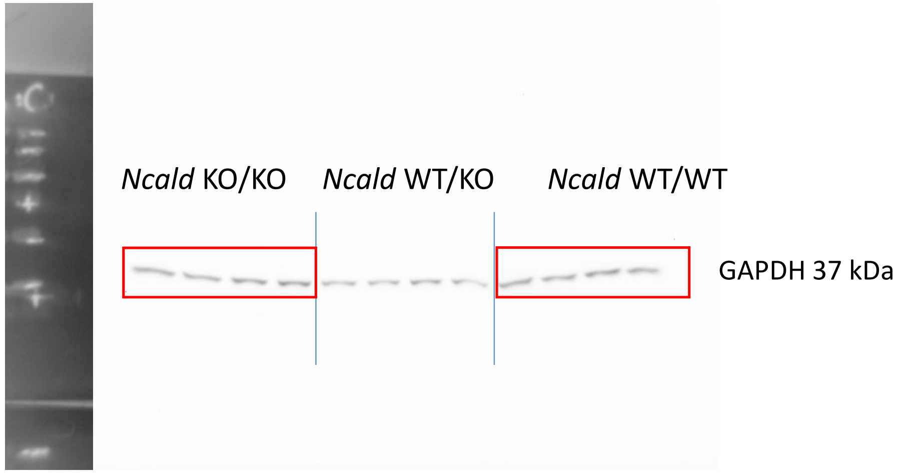

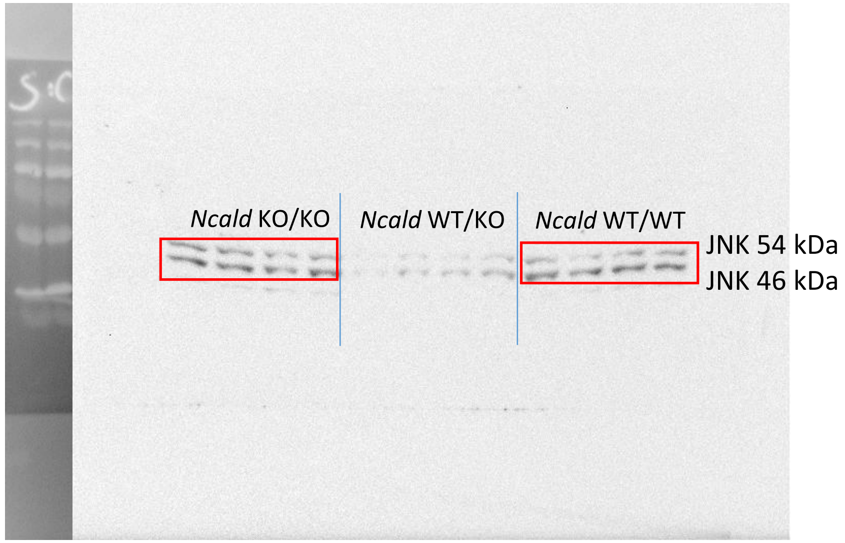

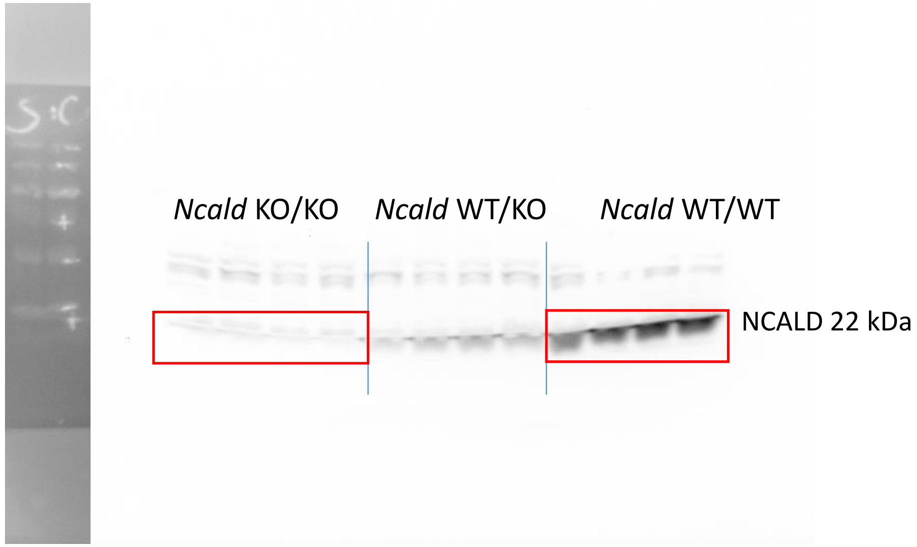

Supplement: Supplementary file 8 [file Data_Sheet_8.PDF]
